# Biosynthesis Investigations of Terpenoid, Alkaloid, and Flavonoid Antimicrobial Agents Derived from Medicinal Plants

**DOI:** 10.3390/antibiotics11101380

**Published:** 2022-10-09

**Authors:** Wenqian Huang, Yingxia Wang, Weisheng Tian, Xiaoxue Cui, Pengfei Tu, Jun Li, Shepo Shi, Xiao Liu

**Affiliations:** 1 Modern Research Center for Traditional Chinese Medicine, Beijing University of Chinese Medicine, Beijing 100029, China; 2 State Key Laboratory of Natural and Biomimetic Drugs, School of Pharmaceutical Sciences, Peking University, Beijing 100191, China

**Keywords:** phytochemicals, antimicrobial agents, biosynthetic pathway, secondary metabolites

## Abstract

The overuse of antibiotics in the past decades has led to the emergence of a large number of drug-resistant microorganisms. In recent years, the infection rate caused by multidrug-resistant microorganisms has been increasing, which has become one of the most challenging problems in modern medicine. Plant-derived secondary metabolites and their derivatives have been identified to display significant antimicrobial abilities with good tolerance and less adverse side effects, potentially having different action mechanisms with antibiotics of microbial origin. Thus, these phyto-antimicrobials have a good prospect in the treatment of multidrug-resistant microorganisms. Terpenoids, alkaloids, and flavonoids made up the predominant part of the currently reported phytochemicals with antimicrobial activities. Synthetic biology research around these compounds is one of the hotspot fields in recent years, which not only has illuminated the biosynthesis pathways of these phyto-antimicrobials but has also offered new methods for their production. In this review, we discuss the biosynthesis investigations of terpenoid, alkaloid, and flavonoid antimicrobial agents—using artemisinin and oleanolic acid (terpenoids), berberine and colchicine (alkaloids), and baicalin (flavonoids) as examples—around their antimicrobial action mechanisms, biosynthesis pathway elucidation, key enzyme identification, and heterologous production, in order to provide useful hints for plant-derived antimicrobial agent discovery and development.

## 1. Introduction

Infectious diseases caused by pathogenic microorganisms are becoming one of the major causes of death worldwide [1]. Antibiotic refers to a chemical substance, with an organic chemical of natural or synthetic origin, that has the capacity to inhibit the growth of and even kill pathogenic bacteria and other micro-organisms [2]. The discovery and development of antibiotics during the 20th century substantially reduced the threat of infectious diseases [3,4]. However, it has been decades since antibiotics with a completely novel mode of action were last delivered to the clinic. Specifically, in the first decade of the 21st century, with the emergence of resistant strains of several important microbials, including *Pneumococci*, *Enterococci*, *Staphylococci*, *Plasmodium falciparum*, and *Mycobacterium tuberculosis* [5], people were faced with this continuing threat on a wider scale than ever before. Multidrug-resistant pathogens are expected to kill about 300 million people prematurely and will have costed the global economy up to USD 100 trillion by 2050 [6,7]. Several factors are involved in the rise of antibiotic resistance, including the existence of efflux pumps, the lack of sensitive antibiotic targets, induction of a stress response of bacterial cells (SOS reaction and RPOS regulation), the transport of drug-resistant genes through the horizontal gene transfer (HGT) mechanism, and the inactivation of antibiotics by hydrolysis or modification, but the main reason is the overuse and misuse of antibiotics in human and animal health and the lack of development of new antibiotics [8,9,10]. Thus, there is an urgent need for new compounds with different mechanisms that can limit antibiotic resistance.

In recent years, people have focused more attention on plants for new antibiotics discovery and development since many experiments have proven that compounds from plants have significant and potentially different antimicrobial effects compared with antibiotics of microbial origin [11,12,13]. The antimicrobial compounds from medicinal plants may inhibit the growth of bacteria, fungi, viruses, and protozoa by different mechanisms than those of presently used antimicrobials and may have a significant clinical value in the treatment of resistant microbial strains [5,14,15]. These agents could perform direct bactericidal action by blocking bacterial DNA synthesis; inhibiting ATPase activity; inhibiting biofilm formation, membrane integrity, or permeability; and resisting the quorum sensing effect [16]. Moreover, many phytochemicals also showed effective antibiotic drug resistance reversal activity, mainly through enzyme modification, plasmid curing, or drug efflux pump [9]. Although some of them do not hold substantial antibacterial potential on their own, their application along with other drugs may considerably augment the antibiotic potential of the drug against which the pathogen was resilient [17]. Moreover, compared with synthetic drugs, plant-derived antibiotics usually have fewer side effects and a lower possibility of drug resistance [10]. On the basis of these advantages, exploring plant-based metabolites is a promising choice to identify new bioactive compounds, which can be used to develop new and effective antimicrobial agents or multidrug-resistant reversal agents.

Secondary plant metabolites are molecules indirectly necessary for the life of plants, which can serve as structural elements or as important tools for plants to adapt to their environment and play a crucial role in many aspects of plant life activities [18]. According to the different chemical structure skeletons and natural origins, plant-derived natural products can be divided into diverse categories. Among them, there is no doubt that terpenoids, alkaloids, and flavonoids compose the dominant part of phytochemicals in the plant kingdom. Moreover, according to a large number of pharmaceutical reports, these three kinds of natural products were also the major source of bioactive antimicrobial candidates’ discovery. This review aims to focus on terpenoid, alkaloid, and flavonoid compounds with antimicrobial activities (including antibacterial, antifungal, antiviral, and/or antiparasitic activities) from medicinal plants, mainly discussing their action mechanisms, biosynthesis pathway elucidation, and biosynthesis key enzyme identification, as well as engineering strain construction.

## 2. Terpenoids

Terpenoids, also known as isoprenoids, are one of the largest natural product families, constituting more than 40,000 primary and secondary metabolites, including monoterpenes (53%), diterpenoids (1%), sesquiterpenes (28%), and others (18%). The basic unit of terpenes is the isoprene unit (C_5_H_8_), which is a simple hydrocarbon. It is the main precursor and could be post-modified through the cytosolic mevalonate (MVA) pathway or the plastid methyl erythritol phosphate (MEP) pathway. Terpenoids are a major source of bioactive natural products. Especially because of their lipophilic characteristics, terpenoids have become one of the major kinds of antimicrobial agents against various microorganisms [19].

### 2.1. The Antimicrobial Mechanisms of Terpenoids

There are mainly five mechanisms through which terpenoids exhibit antimicrobial action according to previous reports.

- Cell membrane destruction: Terpenoids mainly use their lipophilicity to destroy the cell membrane of bacteria. Terpenoids can pass through the phospholipid bilayer of bacteria and diffuse inward, showing antibacterial or bactericidal effects [20]. Since the integrity of the cell membrane is very important for the normal physiological activities of bacteria, the damage of terpenoids to the membrane will affect the bacteria’s basic physiological activities, and the important substances such as proteins and important enzymes in the cell will be lost, finally achieving the antimicrobial effect [21]. It is reported that 1,8-cineole (Table 1), a monoterpene substance extracted from *Eucalyptus globulus Labill*, showed antibacterial effect against *Acinetobacter baumannii, Candida albicans*, a methicillin-resistant *Staphylococcus aureus* (MRSA) strain, and *Escherichia coli* by destroying the cell membrane [22]. In another study, the researchers exposed *Salmonella typhimurium*, *E. coli* O157: H7, *Pseudomonas fluorescence*, *Brochotrix thermophacta*, and *Staphylococcus aureus* cells to cinnamaldehyde (Table 1), carvacrol (Table 1), thymol (Table 1), eugenol (Table 1), and limonene (Table 1), and observed their membrane damage through scanning electron microscopy. These results found that terpenoids can achieve bacteriostatic effects by destroying the membrane structure [23]. The mechanism of action and target sites on microbial cells are graphically illustrated in [20,21].
- Anti-quorum sensing (QS) action: The QS system is an intercellular communication system [20]. It is a communication mode for bacteria to coordinate the interaction between bacteria and other organisms, which is also the main reason for the emergence of antibiotic resistance [19]. The group sensing signal loop of Gram-positive and Gram-negative bacteria has been introduced and illustrated in the literature [24]. Studies have shown that a low concentration of cinnamaldehyde can effectively inhibit the QS effect between bacteria [25]. Low concentrations of carvacrol and thymol can effectively inhibit the self-inducer of bacteria, namely, acyl homoserine lactone (AHL), thus achieving the inhibition of QS [26]. The action mechanism of cinnamaldehyde inhibiting the acyl homoserine lactones and other autoinducers involved in the quorum sensing is illustrated in [27].
- Inhibition of ATP and its enzyme: ATP is the most direct energy source in organisms, and it is also a necessary element for microorganisms to maintain normal operation and work. Terpenoids can act on the cell membrane, resulting in the difference in ATP concentration inside and outside the cell, leading to the disorder of the cell membrane, thus conducting the antibacterial activity [20]. For example, terpenoid eugenol and thymol could target the cell membrane to show fungicidal activity against *C. albicans* by inhibiting H^+^-ATPase, which will lead to intracellular acidification and cell death [28]. In another study, the researchers treated the target pathogen with the MIC of carvacrol. The extracellular ATP concentrations of the samples were measured with the help of a luminometer (Biotek). On the basis of absorbance analysis at 260 nm, this study observed that carvacrol disrupted the *E. coli* membrane, while the release of potassium ions and ATP was also detected [29].
- Inhibition of protein synthesis: The physiological activity of bacteria is inseparable from protein synthesis. Terpenoids, as inhibitors of protein synthesis, can achieve an antibacterial effect by blocking any process of the protein synthesis pathway. Some studies have shown that cinnamaldehyde can reduce the in vitro assembly reaction and the binding reaction of FtsZ (filamenting temperature-sensitive mutant Z)-type protein, a prokaryotic homolog of tubulin that regulates cell division. In addition, cinnamaldehyde can inhibit the hydrolysis of GTP and bind to FtsZ, as well as interfere with the formation of z-loop of cell dynamics, thus showing antibacterial activity against bacteria [30]. In the latest research, the researchers used calculations, biochemistry, and in-vivo-based assays to verify that cinnamaldehyde is a potential inhibitor of *S. typhimurium* (stFtsZ), and its inhibition rate of stFtsZ GTPase activity and polymerization is up to 70% [31].
- The synergistic effect: For example, the synergistic antibacterial effect of eugenol with carvacrol and thymol is due to the ability of carvacrol and thymol to penetrate the extracellular membrane, thus making it easier for eugenol to enter the cytoplasmic membrane or increasing the number, size, and duration of pores to bind to membrane proteins for better antibacterial activity [32]. The reaction mechanism is shown in the literature [27].

### 2.2. Biosynthesis of Terpenoid Precursors

**Figure 1:**
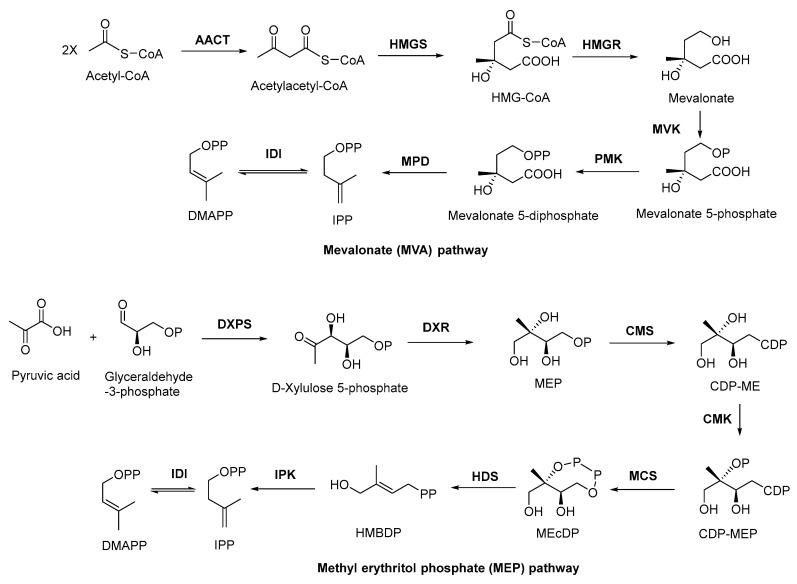
MVA and MEP pathways involved in terpenoid biosynthesis. AACT, acetoacetyl coenzyme A thiolase; HMGS, 3-hydroxy-3-methyl glutaryl coenzyme A synthetase; HMGR, 3-hydroxy-3-methyl glutaryl coenzyme A reductase; MVK, mevalonate kinase; PMK, phosphomevalonate kinase; MPD, mevalonate-5-pyrophosphate decarboxylase; IDI, isopentenyl diphosphate isomerase; DXPS, 1-deoxy-xylose-5-phosphate synthase; DXR, 1-deoxy-xylose-5-phosphate racemic enzyme; CMS, 4-diphoxphocyt-idyl-2-*C*-methyl-2-(E)-butenyl-4-diphosphate synthase; CDP, cytidine-4-diphosphate; CDP-ME, cytidine-4-diphosphate-2-*C*-methylerythritol; CMK, 4-diphoxphocyt-idyl-2-*C*-methyl-D-erythritol kinase; CDP-MEP, cytidine-4-diphosphate-2-*C*-methyl-D-erythritol-2-phosphate; MCS, 2-*C*-methyl-D-erythritol-2,4-cyclodiphosphats synthase; MEcDP, 2-*C*-methyl-D-erythritol-2,4-cyclophosphoric acid; HDS, 1-hydroxy-2-methyl-2-(E)-butenyl-4-diphosphate synthase; HMBDP, 1-hydroxy-2-methyl-2-(E)-butenyl-4-diphosphate; IPK, isopentenyl monophosphate kinase.

There are two important precursors for terpenoid biosynthesis, dimethylallyl pyrophosphates (DMAPP) and isopentenyl diphosphate (IPP), which can both be produced via the MVA or MEP pathways (Figure 1), depending on the organism. The MVA pathway is based on the formation of IPP and DMAPP from acetyl coenzyme A (CoA) through the precursor substance MVA, followed by further condensation of IPP and DMAPP to form sesquiterpenes, triterpenes, and sterols by the action of polyisoprene pyrophosphate synthase. The MEP pathway, on the other hand, is based on pyruvate and glyceraldehyde-3-phosphate in the presence of 1-deoxyxylulose-5-phosphate synthase (DXS) to form DXP. Then, under the catalysis of 1-deoxyxylulose-5-phosphate reductor isomerase (DXR), MEP was formed, followed by further phosphorylation and cyclization to produce IPP, which will be used in the downstream biosynthesis of monoterpenes, diterpenes, and other terpenoids. In plants, both pathways can occur, with the MVA pathway acting in the cytoplasm and the MEP pathway acting in the plastid. In bacteria, terpenoids are generally produced via the MEP pathway, whereas terpenoids are mostly synthesized via the MVA pathway in fungi. Although there are slight differences in the processes of these two pathways, the end products are both DMAPP and IPP [66]. In general, the MEP pathway provides C_5_-pentenyl diphosphate for the synthesis of C_10_ monoterpenes, C_20_ diterpenes, and C_40_ tetraterpenes, while the MVA pathway provides the same generic precursors for the synthesis of C_15_ sesquiterpenes, C_27–29_ sterols, C_30_ triterpenes, and their saponin derivatives [67].

### 2.3. Discovery, Biosynthesis Investigations, and Engineering Strain Construction of the Representative Terpenoid Antimicrobial Agent—Artemisinin

#### 2.3.1. Discovery and Predicted Action Mechanism of Artemisinin

**Figure 2:**
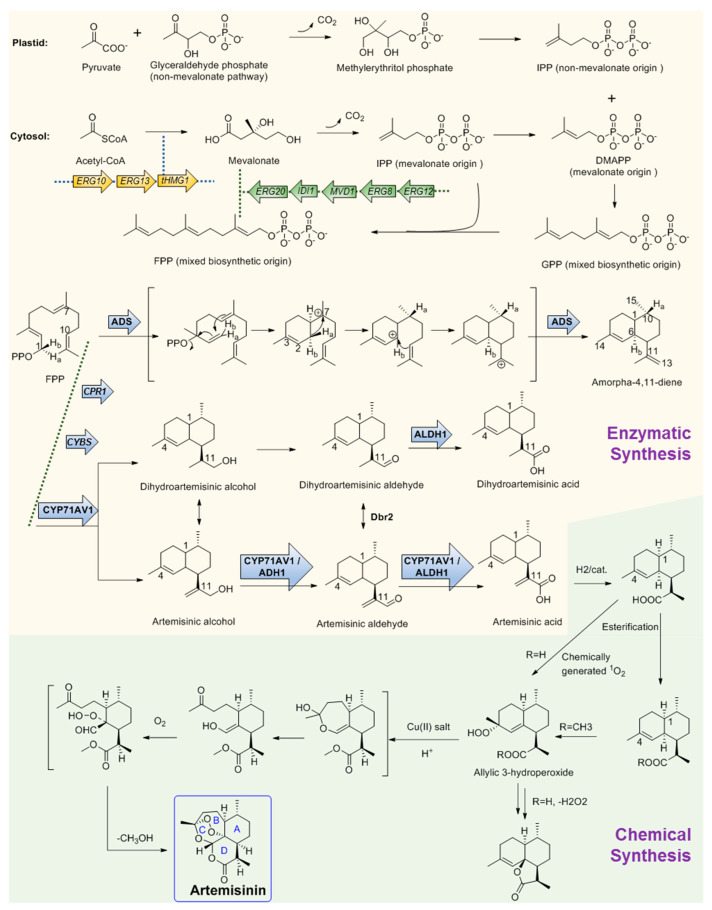
Chemo-enzymatic synthesis of artemisinin. Yellow region shows the biosynthesis pathways for artemisinic acid production. Green region shows the chemical conversion route of artemisinic acid to artemisinin. tHMGR, 3-hydroxy-3-methylglutaryl-coenzyme A reductase; ADS, amorphadiene synthase; CYP71AV1, amorpha-4; 11-diene monooxygenase; ADH1, artemisinic alcohol dehydrogenase; ALDH1, artemisinic aldehyde dehydrogenase 1; Dbr2, artemisinic aldehyde Δ11 (13) reductase; CPR1, cognate reductase of CYP71AV1. Arrows with frames showed the gene elements manipulated by Keasling’s team for artemisinic acid production engineering strain construction.

So far, there have been several reports about terpenoid compounds that displayed desired antimicrobial activities [68]. Among them, the most representative one is undoubtedly artemisinin (Figure 2). Artemisinin (Qinghaosu) is a sesquiterpene endoperoxide isolated from the leaves of the plant *Artemisia annua*, which has a long history of use in traditional Chinese medicine. Malaria, caused by *Plasmodium falciparum*, has been a life-threatening disease for thousands of years [69]. Nowadays, 40% of the world’s population is at risk of malaria infection, and artemisinin is designated as the first-line antimalarial drug by the World Health Organization (WHO). Since the discovery of the antimalarial activities of artemisinin by Chinese scientists in 1971, it has saved millions of lives and represents one of the significant contributions of China to global health. On account of this, the 2015 Nobel Prize for Medicine was awarded to Professor Youyou Tu for her contributions to the discovery and recognition of artemisinin [70].

Although widespread investigations have been carried out, the mechanism of action of artemisinin is still incompletely clarified. It has been widely accepted that the anti-malarial activity of artemisinin is largely dependent on the unusual endoperoxide since derivatives lacking the endoperoxide bridge are discovered to be devoid of antimalarial activity, and the activity could be enhanced by high oxygen tension and by the addition of other free-radical-generating compounds, while some radical scavengers could block the antimalarial activity [71]. Considerable evidence has proven that the killing parasite’s ability of artemisinin-based combination therapies is mediated by free radicals, which are produced from the endoperoxide bridge [72]. The degradation of the endoperoxide bridge in a heme-dependent process could form carbon-centered radicals, which then alkylate multiple targets including heme and proteins at the pathogenic *Plasmodium* blood stage and lead to the conversion of heme to hemozoin and finally lead to the death of the parasite [73].

#### 2.3.2. Key Enzymes Involved in The Biosynthesis Pathway of Artemisinin

The large demand for artemisinin-based combination therapies has caused artemisinin to fall in short supply. To provide more alternative sources, the biosynthesis pathway of artemisinin has been investigated for many years and remarkable achievements have been obtained. Like other regular sesquiterpenes, artemisinin’s biosynthesis precursor is farnesyl pyrophosphate (FPP), which is formed by the condensation of three IPP molecules by either the MVA or plastid non-MVA pathway, respectively [74]. To verify the origin of the precursors, a plant of *A. annua* was grown in an atmosphere containing labeled ^13^CO_2_ for 100 min. Following a chase period of 10 days, artemisinin was isolated and analyzed by ^13^C NMR spectroscopy. The result shows that the precursor IPP can be provided by both the MVA pathway and the non-MVA pathway. As shown in Figure 2, DMAPP was initially provided by MVA origin and then transferred to the plastid, where an IPP unit of non-MVA origin is used for elongation to form geranyl diphosphate (GPP). In the subsequent step, GPP is exported to the cytosolic compartment and converted into FPP using IPP from the MVA pathway [75] (Figure 2). After FPP is formed, the first committed step of artemisinin biosynthesis is the conversion of FPP to amorphadiene by the terpene synthase enzyme amorphadiene synthase (ADS). To explore the catalysis mechanism of ADS, deuterium-labeled FPP at H-1 position was used as the substrate to trace the H-1 hydrogen migration of FPP during cyclization. ^1^H NMR results of amorphadiene showed that one of the hydrogen Ha-1 of FPP migrated to H-10 of amorphadiene, while the other hydrogen Hb-1 remained at its position to label amorphadiene H-6. These observations indicated that ADS may act through an initial formation of a bisabolyl cation intermediate through 1,6-ring closure and one 1,3-hydride shift. Bisabolyl carbocation intermediate would then undergo hydride shift through one direct suprafacial 1,3-shift of axial Ha-1 to C-7 (Figure 2), resulting in the correct *cis*-decalin configuration at C-1 and C-6 of amorphadiene [76,77,78,79]. Following the formation of amorpha-4,11-diene, a cytochrome P450, CYP71AV1, was cloned from *A. annua* and characterized by expression in *Saccharomyces cerevisiae*. CYP71AV1 could catalyze the multiple oxidation steps of amorpha-4,11-diene to produce artemisinic alcohol and artemisinic aldehyde, and finally yield artemisinic acid [80]. In addition, two genes encoding putative artemisinic alcohol dehydrogenase (ADH1) and artemisinic aldehyde dehydrogenase 1 (ALDH1) were characterized from *A. annua* glandular trichomes [81]. ADH1 is a NAD-dependent alcohol dehydrogenase of the medium-chain dehydrogenase/reductase superfamily, with specificity towards artemisinic alcohol. ALDH1 could effectively convert artemisinic aldehyde to artemisinic acid [82].

It is obvious that the Δ11 (13) double bond in amorpha-4,11-diene is reduced during the biosynthesis of artemisinin, which is assumed to occur in artemisinic aldehyde. A corresponding gene, *Dbr2*, was cloned and characterized from *A. annua* [83]. It could specifically reduce artemisinic aldehyde to produce dihydroartemisinic aldehyde, which could be then converted to dihydroartemisinic acid by ALDH1. Further study showed that ALDH1 could also catalyze the oxidation of artemisinic aldehyde as CYP71AV1 did [49]. Conversely, CYP71AV1 cannot catalyze the oxidation of dihydroartemisinic aldehyde. Meanwhile, experimental results showed that there was no direct enzymatic conversion of artemisinic acid into dihydroartemisinic acid. Therefore, there should be two branches that exist during artemisinin biosynthesis [84]. It is well accepted that the primary route is through dihydroartemisinic acid, and the route through artemisinic acid is a side pathway [85,86,87]. From dihydroartemisinic acid, biosynthesis of artemisinin still requires a photooxidative formation of the endoperoxide ring. However, the details of this process, such as the potential involvement of additional enzyme activities, are currently unclear. In 2004, there was a report that, through using the cell-free extracts of *A. annua*, realized the bioconversion of artemisinic acid to artemisinin, but the activity was not observed when using artemisinic acid as the only substrate [88]. Thus, the enzyme in charge of this reaction is still a question. One possibility is that artemisinic acid could be converted into several other compounds such as arteannuin B non-enzymatically, which is later transformed into artemisinin [89]. Another possibility is that dihydroartemisinic acid could undergo rapid plant pigment photosensitized oxidation, followed by subsequent spontaneous oxidation to form artemisinin [90].

#### 2.3.3. Microbial Production of Artemisinic Acid

On the basis of the biosynthesis pathway elucidation, increasingly more attention on artemisinin is now shifting to its microbial production. Particularly represented by Dr. Jay D. Keasling, his team has made great achievements in this field [91]. They combined the biological synthesis of the earlier steps to produce the precursor artemisinic acid and the organic synthetic steps of artemisinic acid to produce artemisinin together and realized the industrial production of semi-synthetic artemisinin for commerce needs. They first constructed the biosynthesis pathway of amorphadiene in *E. coli*. Compared with the expression of DXP pathway genes, a dramatic increase in isoprenoid precursor production was observed when the *S. cerevisiae* MVA pathway was heterologously expressed in *E. coli.* Thus, two plasmids were correspondingly designed. One encoded the MevT operon (known as the ‘top pathway’), which comprises three genes (*atoB*, *ERG13*, and *tHMG1*) that are needed for the conversion of acetyl-CoA to MVA. The second plasmid encoded the MevB operon (known as the ‘bottom pathway’) comprising five genes (*idi*, *ispA*, *MVD1*, *ERG8*, and *ERG12*) for the conversion of MVA to FPP. These two plasmids were subsequently expressed in *E. coli* with the codon-optimized amorphadiene synthase (ADS) gene together. Combined with the optimization of the fermentation conditions, the production of amorphadiene could reach 0.5 g per liter in *E. coli* [92,93,94]. Following this is the next stage: after the identification of CYP71AV1, this project meets a quandary that although the amorphadiene was produced with a higher yield in *E. coli* than in *S. cerevisiae*, *E. coli* is unsuitable for the expression of the P450 enzyme CYP71AV1, which is crucial for the following steps. Thus, in this stage, Keasling’s team switched the expression system of artemisinin to *S. cerevisiae*. Following this, a series of gene manipulations were performed, including: (1) The *S. cerevisiae* strain was engineered to overexpress the MVA pathway, and all genes were integrated into the genome; (2) *ADS* and *CYP71AV1* genes were constructed as plasmid borne; (3) overexpression of a 3-hydroxy-3-methylglutaryl-CoA reductase (tHMGR) occurred to improve the production of amorphadiene; (4) downregulation of *ERG9* occurred, which encodes squalene synthase, catalyzing the first step in the sterol biosynthetic pathway to inhibit the flux from FPP to sterol; (5) a methionine repressible promoter P_*MET3*_ was used to increase amorphadiene production; (6) the *ADS* gene was expressed under the control of the *GAL1* promoter; (7) the *CYP71AV1* gene was expressed along with its cognate reductase (CPR1); (8) yeast strain CEN.PK2 was chosen as the host, which is capable of sporulating sufficiently; (9) every enzyme of the MVA pathway including *ERG20* (the final step for the production of FPP) was overexpressed in CEN.PK2 in an effort to increase the production of amorphadiene; (10) the *GAL80* gene was deleted to ensure constitutive expression of the overexpressed MVA pathway enzymes and the *A. annua*-derived genes; (11) the much cheaper glucose was used as the carbon source instead of galactose; (12) another two enzymes, aldehyde dehydrogenase (ALDH1) and artemisinic alcohol dehydrogenase (ADH1), were combinedly expressed with CYP71AV1, which resulted in the highest production yield of artemisinic acid. With all the above manipulations coupled with the development of the fermentation process, the production of artemisinic acid in the engineering yeast strain was finally as high as 25 g per liter [81,95,96].

#### 2.3.4. Chemical Conversion to Produce Artemisinin

The final stage for artemisinin chemo-enzymatic synthesis is the chemical conversion of artemisinic acid to artemisinin (Figure 2). The chemical process involves a four-step conversion that begins with the reduction of artemisinic acid to dihydroartemisinic acid. Then, the esterification of the carboxylic acid moiety will be performed to block the subsequent formation of side products. The third step is an ‘ene-type’ reaction of the C4–C5 double bond with singlet oxygen (^1^O_2_) to produce an allylic 3-hydroperoxide. Moreover, in the final step, the allylic hydroperoxide undergoes an acid-catalyzed hock fragmentation and rearrangement to afford a ring-opened keto-aldehyde enol. Trapping of this enol with ^3^O_2_ produces a vicinal hydroperoxide aldehyde, followed by a cascade reaction of acid-catalyzed cyclization that could form an endoperoxide bridge to provide artemisinin at last [81]. Finally, through the metabolic engineering of the earlier steps using multiple gene manipulations and following synthetic organic chemistry, the anti-malaria drug artemisinin production system was successfully established and effectively used for industrial production by Sanofi company as the worldwide supplement [91]. Artemisinin is by far the most successful and representative example of the perfect combination of biosynthetic pathway research and industrial production.

### 2.4. Biosynthesis Pathway Investigation of the Terpenoid Antimicrobial Agent—Oleanolic Acid

Oleanolic acid (Table 1) is a pentacyclic triterpenoid originating from a number of medicinal plants. It has desired antimicrobial activity against various bacterial pathogens and viruses [33,97,98,99,100]. Furthermore, the study on this antimicrobial agent is of importance because as a natural source product, there has been no resistance case toward oleanolic acid found yet [101]. The biosynthesis pathway of oleanolic acid has been relatively clear [102].

**Figure 3:**
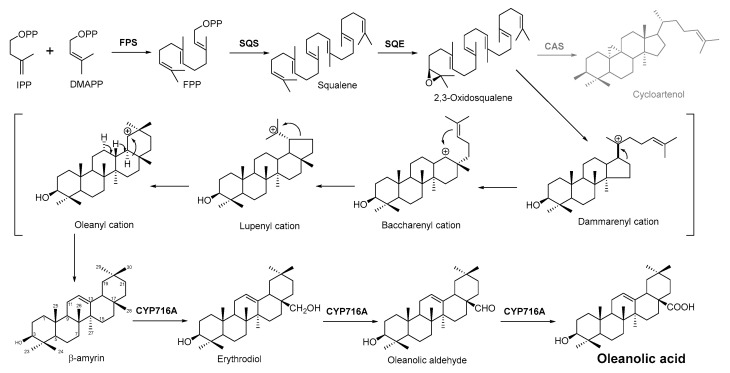
Biosynthesis pathway of terpenoid antimicrobial agent oleanolic acid. FPS, farnesyl diphosphate synthase; SQS, squalene synthase; SQE, squalene epoxidase; CAS, cycloartenol synthase.

In plant cells, acetyl CoA generates DMAPP and IPP through the MVA pathway in the cytosol. IPP and DMAPP are isomerized into FPP under the action of farnesyl pyrophosphate synthase (FPS), and FPP is then converted into squalene under the action of squalene synthase (SQS). Squalene cyclooxygenase (SQE) then oxidizes squalene into a precursor molecule for primary sterol metabolism, 2,3-oxsqualene [103]. From this step, the different cyclizations of 2,3-oxidized squalene become a branching point between primary sterol and secondary triterpene metabolism. For the biosynthesis of plant sterols, the cyclization of 2,3-oxysqualene to the tetracyclic plant sterol precursor cycloartenol is mainly catalyzed by cycloartenol synthase (CAS) [104]. Conversely, the oleanolic acid biosynthetic pathway of our interest, 2,3-oxysqualene, is cyclized by *β*-amyrin synthase (BAS), which was first cloned from the medicinal plant ginseng and subsequently from a variety of other plants [104,105]. This pentacyclic carbon skeleton is assumed to be formed from (3*S*)-2,3-oxidosqualene folded in pre-chair–chair–chair conformation [106]. Opening of the epoxide ring followed by cation–*π* cyclization initially produces a tetracyclic dammarenyl cation. Following ring expansion and the formation of fifth ring, the lupenyl cation is formed [105]. Another ring expansion followed by a series of stereospecific 1,2-hydride shifts and the final abstraction of 12α proton produces β-amyrin [107] (Figure 3). The *C*-28 position of β-amyrin is then oxidized in three consecutive steps by a single cytochrome P450 enzyme, CYP716A12, to produce oleanolic acid. The key enzyme for this step—CYP716A12—was first identified in *Medicago truncatula*, and the study found that erythrodiol, oleanolic aldehyde, and oleanolic acid production were detected in the reaction solution catalyzed by this enzyme [108,109]. Thus, it is suggested that CYP716A12 is a C-28 oxidase of β-amyrin, catalyzing three sequential oxidation reactions of oleanane main chain C-28 rather than a one-step generation. The oleanolic acid biosynthetic pathway is shown in Figure 3.

With the development of synthetic biology, some conventional biosynthetic pathways were interfered with using genetic engineering to improve the target compound’s production. For example, limonene, a cyclic monoterpene of plant origin, is antimicrobially sensitive to *Listeria monocytogenes* and can damage its cell integrity and wall structure [110]. The most classical biosynthetic pathway of limonene is the condensation of IPP and DMAPP to form GPP by the action of geranyl pyrophosphate synthase, and limonene synthase (LS) uses GPP as a substrate to synthesize limonene. However, GPP can also subsequently condense with a molecule of IPP to form FPP, and studies have shown that the synthesis of excessive FPP hinders the efficient synthesis of monoterpenes. According to a recent report, researchers have developed an FPPS mutant (F96W, N127W; FPPS^F96W, N127W^) that can selectively produce GPP without further extension to FPP. In the yeast strain with high isoprene production, fpps^F96W, N127W^ genes were combined with nine plant LS genes, and the *N*-terminal sequence of plasma-membrane-targeted transport peptide (TLS) was truncated. The best effect of 15.5 mg L^−1^ limonene on *Citrus lemon* tls1 (cltls1) was achieved. Moreover, an orthogonal engineering pathway was constructed. In this pathway, limonene could be produced through the condensation of IPP and DMAPP by neryl pyrophosphate (NPP) synthase to form NPP, and limonene synthase can also use NPP as a substrate to synthesize limonene. The expression of *Solanum lycopersicum* nerolidyl diphosphate synthase (SlNDPS1) and *Citrus limon* tLS2 (CltLS2) genes in the same yeast strain made the limonene yield higher than that of traditional methods (28.9 mg L^−1^). Under the action of glucose-induced promoter HXT1, the production of limonene can be increased to more than 900 mg L^−1^ by extensive pathway engineering using the FPPS competitive gene [111].

## 3. Alkaloids

**Figure 4:**
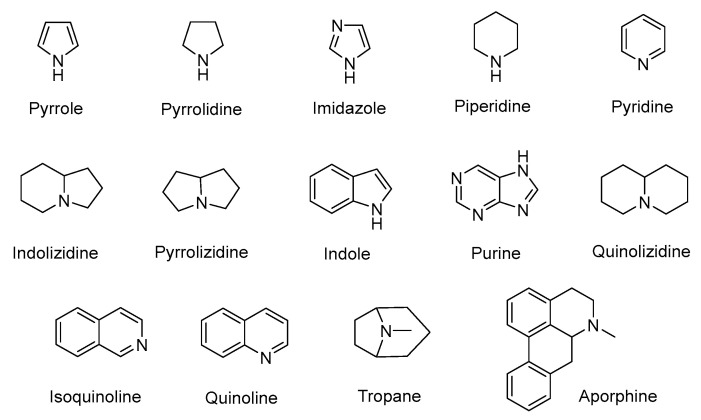
The 14 subgroups of alkaloids.

Alkaloids are a class of structurally diverse nitrogen-containing organic compounds, including more than 20,000 different molecules whose basic nitrogen atom can occur in the form of primary amine (RNH_2_), a secondary amine (R_2_NH), or a tertiary amine (R_3_N) [112]. From the perspective of chemical structure or natural origin, alkaloids can be divided into two broad divisions. The first division contains the non-heterocyclic or atypical alkaloids, also known as protoalkaloids or biological amines, containing nitrogen in the side chain. The second division includes the heterocyclic or typical alkaloids (true alkaloids), containing nitrogen in the heterocycle. Because of their structural complexity, the second division can be further subdivided into 14 subgroups on the basis of the ring structure, as shown in Figure 4 [113].

### 3.1. Plant-Originated Alkaloids with Antimicrobial Bioactivities

Because alkaloids have a proton-accepting nitrogen atom, and one or even more proton-donating amine hydrogen atoms in addition to functional groups, they can easily form hydrogen bonds with proteins, enzymes, and receptors [113]. As a result, alkaloids show a variety of pharmacological activities [8,114,115,116]. Nowadays, there are numerous reports on the antimicrobial activity of plant-derived alkaloids. They could inhibit the growth of fungi, bacteria, viruses, and protozoa through a variety of mechanisms, and may have important clinical value in the treatment of resistant microbial strains [117]. Most alkaloids act as efflux pump inhibitors (EPIs) to exert antimicrobial effects—for example, isoquinoline, protoberberine, quinoline, indole, monoterpene indole, and steroidal alkaloids are reported to be used as competitive inhibitors of efflux pumps in bacteria and fungi [118]. Piperine (Table 1)—a piperidine-type alkaloid—has strong antimicrobial activity against both Gram-positive and -negative bacteria [34], acting as an EPIs in *S. aureus* when combined with ciprofloxacin [35]. Reserpine (Table 1)—an indole alkaloid—is known to be a competitive inhibitor of both primary and secondary active transporter systems. In particular, regarding this latter function, reserpine acts mainly on resistance nodulation division (RND) and the major facilitator superfamily (MFS) [119,120]. In addition, reserpine could reverse Bmr-mediated multidrug resistance by inhibiting drug transport [36,121]. Berberine (Table 1) is a kind of isoquinoline alkaloid. It displays a synergistic effect with the carbapenem antibiotic to re-sensitize imipenem-resistant *Pseudomonas aeruginosa* by inhibiting the MexXY-OprM efflux pump system [37,38,39]. Berberine has shown antibacterial activity against selected oral pathogens and is more effective than saline as an endodontic irrigant against selected endodontic pathogens [122]. Some alkaloids play an antimicrobial role by inhibiting nucleic acid synthesis and repair—for instance, berberine is also an excellent DNA intercalator that accumulates under the drive of cell membrane potential [123]. L-Ephedrine (Table 1), D-pseudoephedrine (Table 1), and L-methylephedrine (Table 1) have antiviral effects on influenza A virus (IAV) in vitro by inhibiting viral replication and altering inflammatory response [40]. Chelerythrine (Table 1), an isoquinoline alkaloid, displays strong antibacterial activity against *S. aureus*, MRSA, and extended-spectrum *β*-lactamase *S. aureus* (ESBLs-SA) through inhibition of cellular division and nucleic acid synthesis [41]. Some alkaloids play an antimicrobial role by changing the permeability of the membrane. 8-Hydroxyquinoline (Table 1) is one of the oldest antibacterial agents [124]. Its high lipophilicity allows it to penetrate bacterial cell membranes to reach its target site of action [42], displaying activity against *S. aureus*, *Haemophilus influenzae*, and *Streptococcus pneumoniae* [43]. Some alkaloids conduct antimicrobial effects by inhibiting the activity of enzymes. For example, michellamine B (Table 1) obtained from the tropical plant *Ancistrocladus korupensis* showed anti-HIV activity by inhibiting the enzymatic activities of reverse transcriptases from both HIV types 1-2 as well as by inhibiting human DNA polymerases α and β [44]. Some alkaloids perform antimicrobial effects by inhibiting the growth of bacteria, such as benzophenanthridine alkaloid sanguinarine (Table 1). It could interfere with Z-ring assembly through inhibiting filamenting temperature-sensitive mutant Z (FtsZ) binding, thus preventing cytokinesis in both Gram-positive and Gram-negative bacteria [45]. Sanguinarine can also affect the binding of FtsZ protofilaments to have a bacteriostatic effect [46].

### 3.2. Biosynthesis Investigation of the Representative Antimicrobial Alkaloid Compound—Berberine

Alkaloids are biosynthetically derived from amino acids such as phenylalanine (Phe), tyrosine (Tyr), tryptophan, ornithine, and lysine. Building blocks from the acetate, shikimate, or deoxyxylulose phosphate pathways are also frequently incorporated into alkaloid structures. Nowadays, the synthetic pathways of multiple kinds of antimicrobial alkaloids have been analyzed and confirmed, such as berberine, colchicine, benzylisoquinoline alkaloids (BIAs), and tropane alkaloids (TAs).

**Figure 5:**
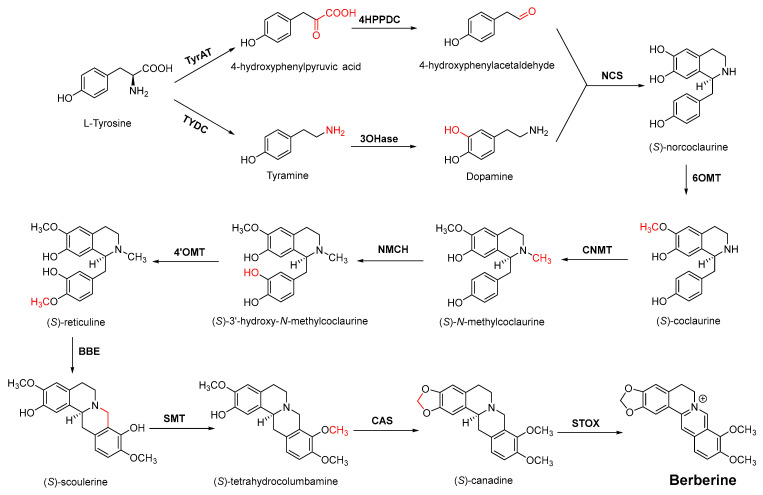
Biosynthesis pathway of the alkaloid antimicrobial agent berberine. TyrAT, L-tyrosine aminotransferase; 4HPPDC, 4-hydroxyphenylpuruvate decarboxylase; TYDC, tyrosine decarboxylase; 3OHase, tyrosine/tyramine 3-hydroxylase; NCS, (*S*)-norcoclaurine synthase; 6OMT, SAM: norcoclaurine 6-*O*-methyltransferase; CNMT, (*S*)-coclaurine *N*-methyltransferase; NMCH, *N*-methylcoclaurine 3′-hydroxylase; 4′OMT, SAM: 3′-hydroxy-*N*-methylcoclaurine 4′-*O*-methyltransferase; BBE, berberine bridge enzyme; SMT, SAM: scoulerine 9-*O*-methyltransferase; CAS, (*S*)-canadine synthase; STOX, (*S*)-tetrahydroprotoberberine oxidase.

Berberine is the main representative quaternary ammonium salt of protoberberines produced from *Berberis* spp. with various antimicrobial activities, especially against Gram-negative bacteria [125,126,127,128]. The generally accepted biosynthesis precursor of berberine is L-Tyr [129]. Biosynthesis from L-Tyr to berberine has 13 steps involving different enzymatic reactions, and all the enzymes involved in this pathway have been biochemically characterized, as shown in Figure 5 [130]. It begins with the formation of the first committed intermediate (*S*)-norcoclaurine, which is formed through the condensation of two Tyr derivatives, dopamine and 4-hydroxyphenylacetaldehyde (4-HPAA). Dopamine and 4-HPAA are synthesized by Tyr decarboxylase (TYDC) and Tyr/tyramine 3-hydroxylase (3OHase), or L-Tyr aminotransferase (TyrAT) and 4-hydroxyphenylpuruvate decarboxylase (4HPPDC), respectively, and they were further condensed by the formation of C-C bonds under the action of (*S*)-norcoclaurine synthase (NCS) to generate the basic 1-benzylisoquinoline core (*S*)-norcoclaurine [131,132,133]. (*S*)-Norcoclaurine continues to be methylated and oxidized to form (*S*)-reticuline, which is a key molecule to derive a series of alkaloids, through four steps under the action of three methyltransferases (*S*-adenosyl-L-methionine (SAM): (*S*)-norcoclaurine 6-*O*-methyltransferase (6OMT) [134,135], SAM: (*S*)-coclaurine-*N*-methyltransferase (CNMT) [136,137], SAM: 3′-hydroxy-*N*-methylcoclaurine 4′-*O*-methyltransferase (4′OMT) [138,139], and one cytochrome P450 enzyme [P450, (S)-*N*-methylcoclaurine 3′-hydroxylase (NMCH)) [140,141,142]. During this biosynthesis, 6OMT catalyzes *O*-methylation at C6 on (*S*)-norcoclaurine to yield (*S*)-coclaurine [135], and (*S*)-coclaurine further undergoes *N*-methylation under the action of CNMT to generate (*S*)-*N*-methylcoclaurine [137]. Then, the P450 enzyme NMCH can convert (*S*)-*N*-methylcoclaurine to (*S*)-3′-hydroxy-*N*-methylcoclaurine [140], and finally it catalyzes the transfer of the *S*-methyl group of SAM to the previous product through 4′OMT to form an important intermediate (*S*)-reticuline base for the synthesis of isoquinoline alkaloids [138]. Subsequently, the key central ring closure is the conversion of the *N*-CH_3_ of (*S*)-reticuline to the berberine bridge carbon, C8 of (*S*)-scoulerine, thereby forming the protoberberine carbon skeleton. This step is accomplished by berberine bridge enzyme (BBE), which is also an important step in the biosynthesis of other isoquinoline alkaloids including protopine, protoberberine, and benzophenanthridine alkaloids [143]. BBE is a key rate-limiting enzyme in the synthesis of (*S*)-scoulerine. More recently, Li et al. [144] achieved high expression of McBBE derived from *Macleaya cordata* in *S. cerevisiae* through codon optimization, *N*-terminal truncation, and CRISPR-Cas9 technology, obtaining a genetically stable *S. cerevisiae* strain with high McBBE expression. Further methylation of (*S*)-scoulerine was performed by *O*-methyltransferase (SAM: scoulerine 9-*O*-methyltransferase (SMT)) [145,146] to yield (*S*)-tetrahydrocolumbamine, which is stereospecifically converted to (*S*)-canadine under formation of the methylenedioxy bridge through (*S*)-canadine synthase [147]. Finally, (*S*)-canadine is oxidatively aromatized to berberine through tetrahydroprotoberberine oxidase [147,148]. The above is the detailed process of berberine biosynthesis from Tyr, and it has been reported that by combining enzymes from the same or different sources, this pathway could successfully synthesize berberine and a series of important intermediates [130,149,150,151] (Figure 5).

### 3.3. Biosynthesis Investigations of the Antimicrobial Alkaloid Compound—Colchicine

Colchicine is an FDA-approved, available, safe, and effective anti-inflammatory drug derived from *Colchicum* and *Gloriosa* species [152,153,154]. On the basis of its unique efficacy as an anti-inflammatory agent, colchicine has been used in the therapy of cardiovascular diseases. Most recently, there have numerous reports suggesting that colchicine could also be used in the treatment of coronavirus disease 2019 (COVID-19) [155,156]. The antiviral activity of this alkaloid is attributed to its ability to bind tubulin dimers and inhibit microtubule assembly, which not only promotes anti-inflammatory effects but also makes colchicine a potent mitotic poison [154,157]. In addition, colchicine may inhibit inflammasome signaling and reduce proinflammatory cytokines, which is a purported mechanism of COVID-19 pneumonia [158].

**Figure 6:**
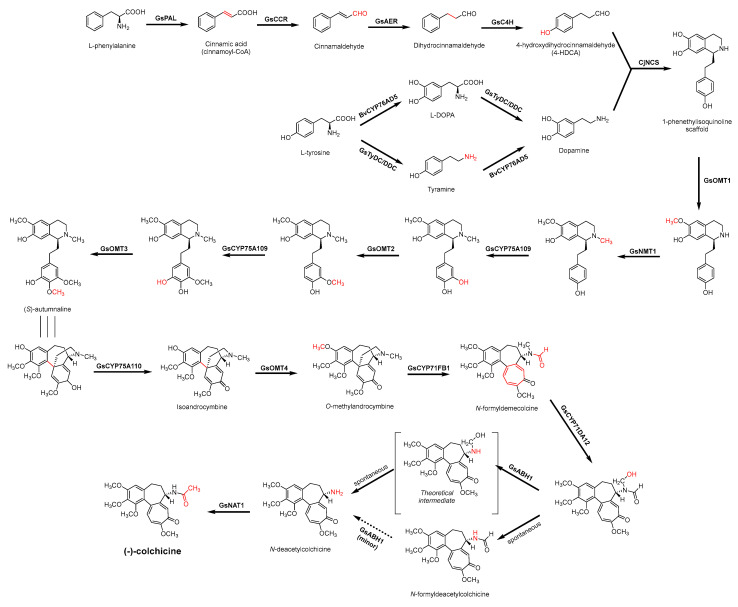
Biosynthesis pathway of alkaloid antimicrobial agent (-)-colchicine. GsPAL, phenylalanine ammonia-lyase; GsCCR, cinnamoyl-CoA reductase; GsAER, alkenal reductase; GsC4H, cinnamate 4-hydroxylase; GsTyDC/DDC, L-tyrosine/L-DOPA decarboxylase; BvCYP76AD5, cytochromes P450 3′-hydroxylase; CjNCS, (*S*)-norcoclaurine synthase; *S*-adenosylmethionine-dependent methyltransferase (MT): GsOMT1, GsOMT2, GsOMT3, GsOMT4, *O*-methyltransferase; GsNMT1, *N*-methyltransferase; GsCYP75A109, GsCYP75A110, GsCYP71FB1, GsCYP71DA12, cytochromes P450; GsABH1, alpha/beta hydrolase; GsNAT1, *N*-acetyltransferase.

For the biosynthesis of colchicine, since Leete conducted the first biosynthetic experiments on colchicine in 1960 [159], the chemical origins of colchicine have been thoroughly studied through an abundance of feeding studies with isotope-labeled substrates in *Colchicum* plants, as well as the structural characterization of colchicine-related alkaloids isolated from species of the Colchicaceae family that helped to define a well-established biosynthetic hypothesis [160,161,162,163] (Figure 6). It has been established that colchicine originated from Phe and Tyr [164]. Similar to the former part of the berberine biosynthetic pathway, the phenethylisoquinoline skeleton of colchicine is also formed by the condensation of an aldehyde with an amine [162]. Namely, the initial amino acids Phe and Tyr are processed into 4-hydroxydihydrocinnamaldehyde (4-HDCA) and dopamine, respectively, which are joined through a Pictet–Spengler reaction to form a 1-phenethylisoquinoline scaffold [162,163,165]. The scaffold then undergoes a series of methylations and phenyl ring hydroxylations to yield (*S*)-autumnaline [163], which proceeds to *para–para* phenol coupling to create a bridged tetracycle [166]. An unusual oxidative ring expansion followed, yielding the characteristic tropolone ring of the colchicine carbon scaffold, which is essential for the tubulin-binding activity of colchicine [167]. The biosynthesis of colchicine is further accomplished through final processing and *N*-acetylation of the extruded nitrogen atom [168]. 

### 3.4. De Novo Biosynthetic Production of Colchicine in Nicotiana benthamiana

In view of the above research, Sattely et al. [169,170] established a metabolic pathway of tropolone-containing colchicine alkaloids by using a combination of transcriptomics, metabolic logic, and pathway reconstitution. The first stage is the generation of the key precursor 1-phenethylisoquinoline scaffold, which requires the Pictet–Spengler condensation of 4-HDCA and dopamine derived from the amino acids Phe and Tyr. Labeling studies have shown that 4-HDCA is produced from Phe through a metabolic pathway analogous to the biosynthesis of monolignols [164,171]. Through hierarchical clustering analysis of *Gloriosa superba* transcriptomic data utilizing the other identified colchicine biosynthesis genes, the researchers demonstrated co-clustering of many monolignol biosynthetic gene orthologs (GsPAL, Gs4CL, GsCCR, GsAER, GsC4H, and GsDAHPS), and their heterologous co-expression in *N. benthamiana* resulted in the production of 4-HDCA. For dopamine formation, the incorporation of L-Tyr and tyramine into colchicine demonstrated the activity of L-Tyr/L-DOPA decarboxylase (TyDC/DDC) and 3′-hydroxylase enzymes [161]. The researchers identified a TyDC/DDC homolog (GsTyDC/DDC) highly co-expressed with other identified colchicine biosynthesis genes in the public *G. superba* transcriptome via a similar analysis approach, combining it with 3′-hydroxylase BvCYP76AD5 from *Beta vulgaris* to produce L-DOPA successfully [169,172]. Furthermore, the modified (*S*)-norcoclaurine synthase from *Coptis japonica* (CjNCS) was utilized to catalyze the condensation of 4-HDCA with dopamine to produce the first alkaloidal precursor 1-phenethylisoquinoline. The NCS is a previously characterized plant Pictet–Spenglerase, which condenses 4HPAA and dopamine within the biosynthesis of benzylisoquinoline alkaloid (BIA) [173]. It can also condense a wide range of aldehyde substrates with dopamine [174,175]. The precursor will yield (*S*)-autumnaline by further modification (hydroxylations, methylations). (*S*)-Autumnaline then undergoes enzyme-catalyzed phenolic coupling together with further modification to produce *O*-methylandrocymbine, which is then converted to colchicine via homoallylic ring expansion [176,177]. On the basis of the above information, the researchers utilized eight genes (GsOMT1, GsNMT1, GsCYP75A109, GsOMT2, GsOMT3, GsCYP75A110, GsOMT4, and GsCYP71FB1) explored from *G. superba* to act on 1-phenethylisoquinoline for the biosynthesis of the colchicine precursor *N*-formyldemecolcine, which contains the characteristic tropolone ring and pharmacophore of colchicine. Combining all the above genes, the authors engineered a biosynthetic pathway (16 enzymes in total) in *N. benthamiana* and realized the de novo biosynthetic production of *N*-formyldemecolcine starting from amino acids Phe and Tyr in the commonly used model plant [169]. Subsequently, enzymes that catalyze the *N*-demethylation, *N*-deformylation, and *N*-acetylation (GsCYP71FB1, GsABH1, GsNAT1) of *N*-formyldemecolcine were further excavated and transferred into *N. benthamiana*. Ultimately, through the heterologous system of 20 genes from *G. superba* (17 genes) and other plants (3 genes), total biosynthesis of enantiopure (-)-colchicine was successfully achieved from primary metabolites [170] (Figure 6).

### 3.5. Biosynthesis Investigations of Other Antimicrobial Alkaloids

As a major source of bioactive natural products, in addition to the above-discussed berberine and colchicine, there are still many alkaloids that showed desirable antimicrobial activities whose biosynthesis pathways have also been clarified. Quinoline alkaloids such as 8-hydroxyquinoline are important kinds of nitrogen-containing heterocyclic aromatic compounds with a broad range of antimalarial, antibacterial, antifungal, and antiviral activities. Quinoline alkaloids mainly exist in the Rutaceae family, and their biosynthesis is derived from 3-hydroxyanthranilic acid, a metabolite formed through a series of enzymatic reactions of tryptophan. Specifically, 3-hydroxyanthranilic acid and malonyl-SCoA are condensed and then cyclized to yield quinoline alkaloids [178]. Monoterpenoid indole alkaloids (MIAs)—a large group of natural products derived from plants, such as camptothecin, quinine, and vinblastine—exhibited anticancer, antimalarial, and antibacterial effects [179,180]. Secologanin is the terminus of the monoterpenoid biosynthesis branch and is coupled to tryptamine by strictosidine synthase (STR) to form strictosidine, which is the universal MIAs precursor in plants. The tomato plant, *S. lycopersicum* L., produces the cholesterol-derived steroidal alkaloids tomatine and tomatidine. Tomatidine selectively and potently inhibits small-colony variants of *S. aureus* that cause opportunistic infections in patients with cystic fibrosis [181], and also has potent fungistatic activity against *Candida* spp. with low toxicity to human cells [182]. Their biosynthesis begins from the precursor dehydrotomatidine via enzymatic dehydrogenation, isomerization, and sequential reductions [183]. Scopolamine is a kind of TA that is present in many different plants of the Solanaceae family and is classified as essential medicine by the WHO. Scopolamine showed considerable antifungal activity [184,185]. Smolke et al. [186] realized the construction of a modular biosynthetic pathway by engineered baker’s yeast for the production of medicinal TA scopolamine, starting from simple sugars and amino acids. Genetic-level manipulations they performed included functional genomics to identify missing pathway enzymes, protein engineering to enable expression of functional acyltransferases through trafficking to the vacuole, heterologous transporters to facilitate intracellular routing, and strain optimization to increase titers. 

The enormous potential of alkaloids as drug precursors is far from exhausted, and various pharmacological effects continue to be reported and reviewed [187]. In addition, emerging biotechnologies have been optimized for plants, including metabolomics, CRISPR-based gene editing, and heterologous yeast platforms, enabling the production of diverse and complex plant compounds. It is reasonable to expect that with an increased understanding of the biosynthesis of other antimicrobial alkaloids, increasingly more alkaloid antimicrobial agents could be explored and mass produced in the near future.

## 4. Flavonoids

Flavonoids widely exist in plants, being the general name of a series of compounds derived from 2-phenyl chromogenic ketones. According to the chemical properties, positions, and types of substituents on the ring, flavonoids can be divided into several subclasses, such as flavones, flavonols, dihydroflavones, dihydroflavonols, isoflavones, chalcone, aurone, and anthocyanidin, among others [188]. The abundance and diversity of chemical structures of flavonoids determined their wide-spectrum biological activities. In addition to the traditional antioxidant, anti-radiation, radicals scavenging, anti-inflammatory, and anti-tumor activities, flavonoids are also reported to possess remarkable antimicrobial bioactivities [189]. They could effectively inhibit bacteria, viruses, and fungi, having good therapeutic effects on infections caused by various pathogenic microorganisms, including *S. aureus*, *Bacillus subtilis*, *P. aeruginosa*, *E. coli*, *S. typhimurium*, *C. albicans*, and *Aspergillus flavus*. These compounds are not easy to produce drug resistance and have high clinical therapeutic values. For example, oral candidiasis is one of the most common types of oral mucosal infection caused by the yeast-like fungus *Candida*. The elderly and children with low immunity are very susceptible to infection. Phloretin (Table 1) can inhibit the pathogenicity and virulence factors of *C. albicans* both in vivo and in vitro, and is considered to be an effective candidate for the treatment of oral candidiasis [64]. Other flavonoids such as apigenin (Table 1) and quercetin (Table 1) have been proven to have significant antibacterial and antiviral activities [190,191]. Quercetin, when taken together with vitamin C, is helpful to prevent and treat patients with early respiratory tract infections. According to the report, when quercetin is used for phytotherapy, patients with mild COVID-19 symptoms have a shorter time to clear the virus [192]. Thus, the plant-originated flavonoids can be used as an ideal natural source to explore novel antimicrobial agents [193]. 

### 4.1. Structure–Activity Relationship Study on Antimicrobial Activity of Flavonoids

The antibacterial activity of flavonoids has attracted extensive attention from researchers. Correspondingly, the relationship between the chemical structure and biological activity has been discussed in depth. It was found that the antibacterial activity of flavonoids was mainly related to the existence of hydroxyl groups on the aromatic skeletons of flavonoids and the types of substituents. In particular, flavonoids substituted by hydrophobic groups, such as propenyl, acyl, alkyl amino chain, alkyl chain, and nitrogen-containing or oxygen-containing heterocyclic groups, have been proven to have better antibacterial potential [194]. Smejkal et al. [195] tested the antibacterial activities of eight flavonoids isolated from *Paulownia tomentosa* towards *S. aureus*. The results showed that hydroxylation at the C-5 position of ring A was very important to enhance the antibacterial activity of flavonoids. The in vitro antibacterial activities of a variety of chalcone derivatives towards MSSA and MRSA were tested. The results showed that chalcones with hydroxyl substitution at the 2 or 4 positions of the B ring had antibacterial activity. However, the methylation of the active hydroxyl groups generally eliminated or weakened its antibacterial activity [196]. Celiz et al. [52] found that acylated flavonoid derivatives usually showed a high inhibitory effect on Gram-positive bacteria. For example, the activity of hesperidin (Table 1) against *S. aureus* and *L. monocytogenes* can be greatly increased by connecting the saturated fatty chain containing 10-12 carbon atoms to the ring of hesperidin. Similar results were also obtained by Babu et al. [54]. They found that the introduction of the acyl group at the C-7 position of oroxylin A (Table 1) can significantly enhance the antibacterial activity. When the acyl group contains long-chain alkyl or phenyl, derivatives of oroxylin A showed even stronger antibacterial activity. Moreover, the antibacterial potential of nitrogen-containing flavone derivatives was also investigated. It was found that the antibacterial activity of nitrogen-substituted apigenin derivatives was much higher than that of apigenin [55].

### 4.2. Antibacterial Effects and Action Mechanisms of Flavonoid Antimicrobial Agents

The antimicrobial mechanism of flavonoids mainly includes the following aspects: inhibiting the energy metabolism of bacteria, interfering with the cell wall of bacteria, destroying the integrity of the cell membrane and increasing its permeability, inhibiting bacterial efflux pumps, inhibiting the metabolism of bacterial nuclear acid, inhibiting bacterial mobility, and reducing the expression of virulence factors to weaken the pathogenicity [194]. Chinnam et al. [56] reported that flavonoids such as hesperidin, morin (Table 1), and silymarin (Table 1) can inhibit the F_1_F_o_ ATPase activity of *E. coli* by binding to the polyphenol binding bag of ATP synthase, thus inhibiting the energy metabolism of bacteria and further exerting the bacteriostatic effect. Navarro-Martinez found for the first time that the epigallocatechin gallate (Table 1) in green tea has strong antibacterial activity against *Stenotrophomonas maltophilia*, mainly by inhibiting the dihydrofolate reductase of *S. maltophilia* [57]. The flavonol compound quercetin could inhibit the growth of various drug-resistant microorganisms. It can suppress the herpesvirus and poliovirus by inhibiting viral polymerase and viral nucleic acid. The flavonol compound galangin (Table 1) could directly destroy the plasma membrane or weaken the cell wall of *S. aureus*, which will lead to osmotic lysis, thus resulting in a bacteriostatic effect [59]. Catechin (Table 1), a flavonoid in green tea, could inhibit the bacterial DNA gyrase by binding to the ATP binding site of the B subunit of the bacterial gyrase, the inactivation of which will cause the death of the bacteria [60]. Studies have shown that bacteria can migrate on semi-solid surfaces in a flagella-driven manner, and this coordinated movement form is considered to be related to the antibiotic resistance of various human pathogens [197]. Pejin et al. [58] discussed the antibacterial mechanism of catechin, caffeic acid, quercetin, and morin against *P. aeruginosa PAO1*. The results showed that quercetin could inhibit the formation of its biofilm at 0.5 MIC concentration. *P. aeruginosa* biofilm formation also depends on the flagellum (swimming motility) and type IV pili (twitching motility). Moreover, among the four compounds tested, quercetin was the only one found to effectively reduce the convulsive movement of *P. aeruginosa*. Fathima et al. [61] used the Gram-positive bacteria *B. subtilis* and Gram-negative bacteria *E. coli* as model organisms to prove that catechins play an antibacterial role mainly by producing active oxygen to cause bacterial cell membrane rupture. Wang et al. [65] reported that silybin (Table 1), a flavonoid compound, combined with ciprofloxacin can improve the antibacterial efficiency by inhibiting the efflux pump of MRSA. Liu et al. [64] established a mouse oral candidiasis model to explore the inhibitory effect of phloretin on the pathogenicity of *Candida albicans*. The results show that phloretin can eliminate virulence factors in vitro, such as inhibition of biofilm formation, yeast-to-hyphae transition, and secretion of protease and phospholipase, in order to play an inhibitory role.

### 4.3. Plant Type III Polyketide Synthase

**Figure 7:**
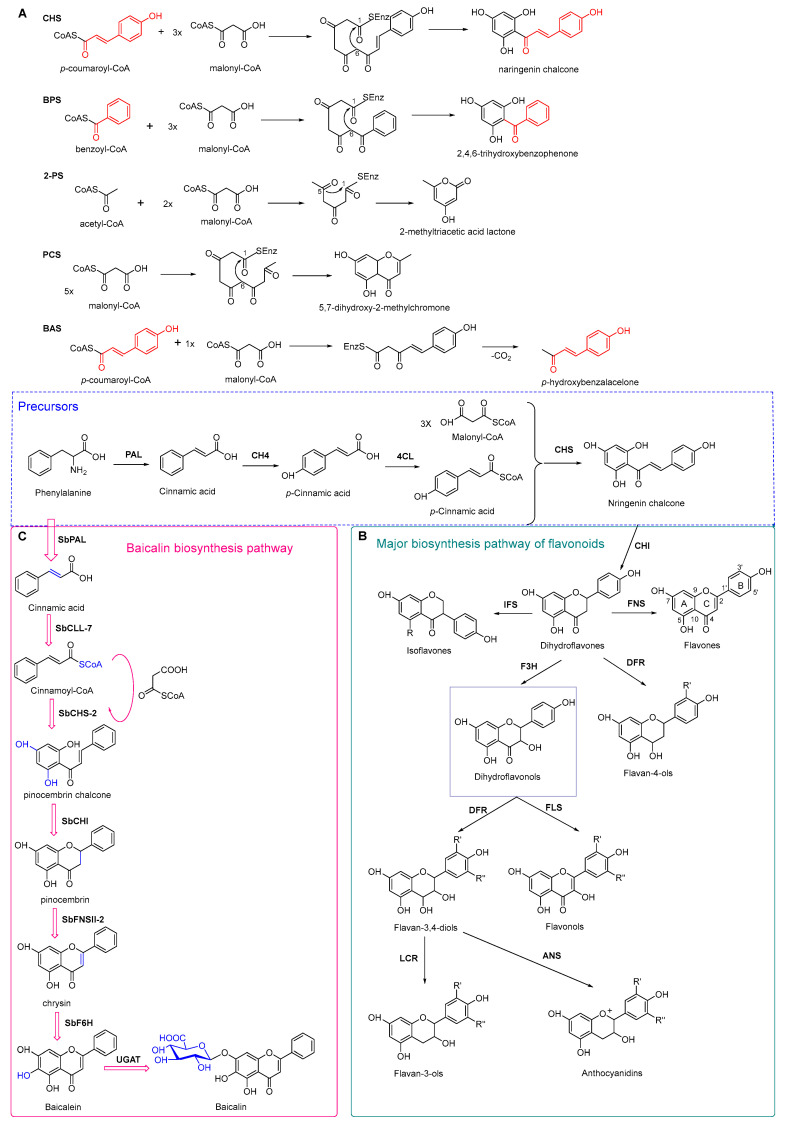
(**A**) Typical reactions catalyzed by plant type III PKSs. (**B**) Main skeleton types of plant-derived flavonoid compounds. (**C**) Biosynthetic pathway of flavonoid antimicrobial agent baicalin. CHS, chalcone syntheses; BPS, benzophenone synthase; 2-PS, 2-pyrone syntheses; PCS, pentaketide chromone synthase; BAS, benzalacetone synthase; PAL, phenylalanine ammonia lyase; CH4, cinnamic acid 4-hydroxylase; 4CL, 4-coumarin CoA ligase; CHS, chalcone synthase; CHI, chalcone isomerase; IFS, isoflavone synthase; FNS, flavone synthase; F3H, flavanone 3-hydroxylase; DFR, dihydroflavonol 4-reductase; FLS, flavonol synthase; ANS, anthocyanidin synthase; LCR, leucoanthocyanidin reductase; SbCLL-7, cinnamate CoA ligase; SbCHS-2, pinocembrin chalcone synthase; SbFNS II-2, flavone synthase; SbF6H, flavone 6 hydroxylase; UGAT, UDP-glucuronic acid transferase [188,204].

The key enzyme involved in the biosynthesis of flavonoids in plants is type III polyketide synthase (PKS), which not only is the key rate-limiting enzyme in the biosynthesis and metabolism pathway of flavonoids but also determines the formation of the basic molecular skeleton of these compounds. Plant type III PKS can repeatedly catalyze the initiation, extension, and cyclization reactions to form polyketone products. At present, nearly 30 plant PKSs genes with different functions have been successively explored and verified in the biosynthetic pathway of various polyketides, such as chalcone syntheses (CHS), benzophenone synthase (BPS), 2-pyrone syntheses (2-PS), pentaketide chromone synthase (PCS), and benzalacetone synthase (BAS). Among them, CHS is involved in the synthesis of all flavonoids of plant origin and has been deeply studied [198]. This enzyme was first cloned from parsley. It could catalyze the three acetyl groups of malonyl CoA to be connected to the molecule of 4-coumaroyl CoA through a continuous condensation reaction, and then generate naringenin chalcone through Claisen-type cyclization reaction, which is the critical intermediate for the biosynthesis of many flavonoid compounds [199], and will be then converted to various flavonoid compounds by downstream tailoring enzymes such as chalcone isomerase (CHI), flavanone 3-hydroxylase (F3H), flavone synthase (FNS), flavonol synthase (FLS), isoflavone synthase (IFS), and polyketide reductase [200] (Figure 7). Since then, researchers have isolated hundreds of CHS genes from lily, rice, corn, alfalfa, and other plants. The protein structure and catalysis mechanism of CHS have also been elucidated [200]. In 1999, Ferrer and his colleagues reported the X-ray crystal structure of *Medicago sativa* CHS2 at 1.56 Å resolution [201]. Through crystallography and site-directed mutagenesis, it was clarified that the key amino acid residues that determined CHS catalytic activities were Cys164, His303, and Asn336 [198]. During the formation of flavonoids, Cys164 acts as a nucleophilic active site and an attachment site for intermediates, while His303 and Asn336 play an important role in the decarboxylation of malonyl CoA. In addition to the key ternary amino acid residues, its internal active site also includes a coenzyme, a binding tunnel, a promoter-substrate-binding pocket, and a cyclization pocket. In recent years, with the elucidation of flavonoids’ biosynthetic metabolic pathway and the development of synthetic biology, it is possible to obtain large-scale flavonoids by building microbial cell factories. *E. coli* and *S. cerevisiae* are common microbial hosts. Genetic engineering strategies such as optimization of culture conditions, modular co-culture technology, and iterative high-throughput screening methods have been used in the construction and improvement of the engineering strains to obtain high-yield target compounds [202,203].

### 4.4. Biosynthesis Investigations of the Antimicrobial Flavonoid Compound—Baicalin

Baicalin (Table 1) is one of the representative flavonoid antimicrobial agents isolated from *Scutellaria baicalensis*. It has been applied as a natural antibacterial agent against foodborne pathogens such as *Salmonella* and *Staphylococcus* spp. [62]. Moreover, this compound also showed significant anti-HIV-1 activity as a nonnucleoside reverse transcriptase inhibitor [205]. Meanwhile, it can prevent the entry of HIV-1 into animal cells by perturbing the interaction between HIV-1 Env protein and HIV-1 co-receptors on the cell surface [206]. As one of the popular lead natural products from medicinal plants for preventing HIV infection, the biosynthesis pathway of baicalin has been fully analyzed, as shown in Figure 7 [188,204]. There are two different biosynthetic metabolic pathways existing in *S. baicalensis*, namely, the aerial flavone part pathway and the root-specific flavone pathway. Baicalein, the precursor of baicalin, is synthesized through the root-specific flavone pathway. The reaction process is as follows: amino acid Phe was used as a biosynthesis precursor that could generate cinnamic acid under the action of SbPAL. Subsequently, cinnamic acid forms cinnamoyl CoA under the catalysis of cinnamate CoA ligase (SbCLL-7). Then, pinocembrin chalcone synthase SbCHS-2 will catalyze cinnamoyl CoA to generate pinocembrin chalcone, which will be further converted to dihydroflavone pinocembrin by SbChI. Next, pinocembrin could be catalyzed by flavone synthase (SbFNSII-2) to form chrysin, finally leading to the production of baicalein under the catalysis of flavone 6-hydroxylase (SbF6H) [207]. As the 7-*O*-glucuronic acid product of baicalein, baicalin could be biosynthesized by UDP glycosyltransferase (UGT) to transfer glucuronic acid to the 7-hydroxyl group of baicalein. Pei et al. [208] identified the baicalin metabolic accumulation pattern and tissue-specific expression patterns of a total of 124 UGTs in *S. baicalensis*. Combined with phylogenetic analysis, four SbUGAT genes were screened out to be able to use UDP-glucuronic acid as a sugar donor to catalyze the conversion of baicalein to baicalin. On the basis of the illumination of the biosynthesis pathway, heterologous production of baicalin has been successfully realized in both *E. coli* [209] and model plant *Lycopersicon esculentum* [210].

## 5. Conclusions and Perspective

Currently, plant-derived antimicrobial agents are still in the early stage of research. The developed plants only account for a very small number of global plants. In addition, the potential synergy or antagonism between plant compounds and antibiotics is still uncertain. Moreover, there is no research showing the resistance of plant antimicrobial agents, so whether there is resistance is unknown. Moreover, some plant compounds have not been tested to prove their effectiveness and safety. Furthermore, studies on the mechanisms of action, exploring the potential synergistic or antagonistic effects and improving the bioavailability, stability, and physicochemical property of the candidate compounds, were also very important before their clinic uses.

Terpenoids, alkaloids, and flavonoids made up the predominant part of the currently reported phytochemicals with antimicrobial activities. Synthetic biology research around these compounds is one of the hotspot fields in recent years. It can be seen that even for artemisinin—one of the most famous antimalaria drugs with in-depth biosynthesis investigation—its whole biosynthesis pathway still has several key enzymes to be discovered, let alone many plant-originated compounds whose biosynthesis pathway is obscure. Thus, a lot of challenges have remained in the investigations of plant-derived compounds. 

In our opinion, one of the greatest challenges may be the discovery of genes because normally functional genes in plants are not clustered. Although recently there are reports that found that the co-expression of physically linked genes occurs frequently, it is still very difficult to explore a new gene through gene cluster searching, especially considering the limited number of plant species with the known whole-genome sequence. In addition, plants usually have different organs and tissues, and the gene expression level could be tissue specific, which makes the selection of the gene extraction material more complex. Moreover, genes in plants often exist in homologs, and their expression could be different and affected by multiple factors such as the environment, living position, temperature, and developmental stages. Currently, the frequently used method for gene discovery in plants is homology-based cloning. However, it is hard to realize in those enzymes with new functions or that do not have enough known templates. With the rapid development of sequencing and transcriptomic advances, enzymes can be discovered by the ‘omics’ tools, such as genomics, transcriptomics, proteomics, and metabolomics. By comparing transcriptome, proteome, and metabolome data from different conditions, candidate genes could be selected and subsequently tested for their putative activity—this approach has been successfully used in the discovery of a large number of unknown genes in phytochemical substances’ biosynthesis pathways. 

The other important field that synthetic biology focuses on is the biosynthetic pathways reconstructing and optimizing the production of secondary metabolites. Recent plant genome editing/engineering methods such as transcription activator-like effector nucleases (TALENS), zinc-finger nucleases (ZFNs), and CRISPR-Cas open new avenues for ration design of the biosynthesis pathway. Using these approaches, genes of interest could be constructed in a highly effective way; meanwhile, the side pathways could be eliminated to a large extent. The targeted antimicrobial or resistance–reversal agents could be produced in the transgenic microorganisms or plants, which have had great success in the production of artemisinin [91], as well as other valuable compounds such as vinblastine [211,212], etoposide aglycone [213], vindoline, and catharanthine [212]. Lastly, with the continuous discovery of new phytochemicals, deep clarification of pharmacological mechanisms, and comprehensive understanding of specific biosynthesis pathways, plant-derived natural products will become increasingly more useful therapeutic antimicrobial candidates in the future.

## Figures and Tables

**Table 1:** Summary of the antimicrobial effects of some plant-derived terpenoids, alkaloids, and flavonoids.

	Compounds	Chemical Structures	Target Microorganisms	Antimicrobial Effects	Reference
Terpenoids	1,8-cineole	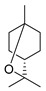	*A. baumannii* *C. albicans* MRSA strain *E. coli*	Cell membrane destruction	[22]
	cinnamaldehyde	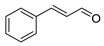	*S. typhimurium* *E. coli* O157: H7 *P. fluorescence* *B. thermophacta* *S. aureus*	1. Cell membrane destruction 2. Anti-quorum sensing action 3. Inhibition of protein synthesis	[23,25,30,31]
	carvacrol	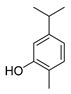	*S. typhimurium* *E. coli* O157: H7 *P. fluorescence* *B. thermophacta* *S. aureus* *P. fluorescens* KM121	1. Cell membrane destruction 2. Anti-quorum sensing action 3. Inhibition of nucleic acid synthesis 4. The synergistic effect 5. Inhibits cell movement and bacterial invasion	[23,26,27,29,32]
	thymol	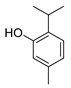	*S. typhimurium* *E. coli* O157: H7 *P. fluorescence* *B. thermophacta* *S. aureus* *P. fluorescens* KM121	1. Cell membrane destruction 2. Anti-quorum sensing action 3. Inhibition of nucleic acid synthesis 4. The synergistic effect	[23,26,27,28,32]
	eugenol	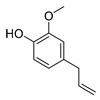	*S. typhimurium* *E. coli* O157: H7 *P. fluorescence* *B. thermophacta* *S. aureus*	1. Cell membrane destruction 2. Inhibition of nucleic acid synthesis 3. The synergistic effect	[23,27,28,32]
	limonene	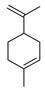	*A. baumannii* *C. albicans* MRSA strain *E. coli*	Cell membrane destruction	[23]
	oleanolic acid	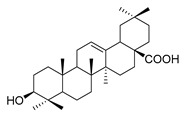	*E. coli* *S. aureus* *Enterococcus faecalis* *P. aeruginosa*	Antibacterial	[33]
Alkaloids	piperine	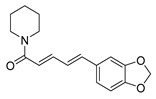	*S. aureus* *B. subtilis* *Salmonella* sp. *E. coli*	Efflux pump inhibition	[34,35]
	reserpine	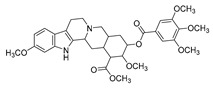	*E. coli*	Efflux pump inhibition	[36]
	berberine	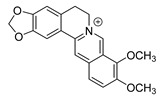	*E. coli* *Micrococcus luteus* *P. aeruginosa* *Prevotella intermedia* *Fusobacterium nucleatum* MRSA strain	1. Efflux pump inhibition 2. DNA-intercalating 3. Growth inhibition	[37,38,39]
	*L*-ephedrine	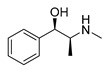	Influenza A virus	DNA-intercalating	[40]
	*D*-pseudoephedrine	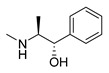	Influenza A virus	DNA-intercalating	[40]
	*L*-methylephedrine	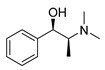	Influenza A virus	DNA-intercalating	[40]
	chelerythrine	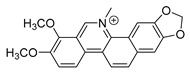	*S. aureus* MRSA strain ESBLs-SA	1. Nucleic acid synthesis and repair inhibition 2. Growth inhibition	[41]
	8-hydroxy quinoline	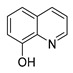	*S. aureus* *H. influenza* *S. pneumoniae*	Permeability change of membrane	[42,43]
	michellamine b	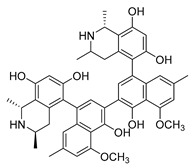	HIV	Protein activity inhibition	[44]
	sanguinarine	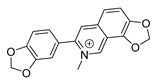	*K. pneumoniae* MRSA strain *P. aeruginosa* *Streptococcus pyogenes*	1. DNA-intercalating 2. Growth inhibition	[45,46]
	roemerine	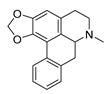	*S. aureus* *B. subtilis*	1. Efflux pump inhibition 2. Permeability change of membrane	[47,48]
	dihydrochelerythrine	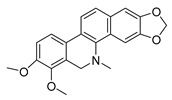	*S. aureus* MRSA strain	Growth inhibition	[49]
	evodiamine	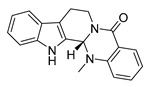	*M. tubercolosis*	Peptidoglycan biosynthesis inhibitor	[50,51]
Flavonoids	hesperidin	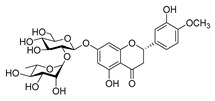	*S. aureus* *L. monocytogenes*	Inhibit bacterial growth by modulating the expression of virulence factors	[52] [53]
	oroxylin a	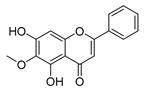	*B. subtilis* *S. aureus*	/	[54]
	apigenin	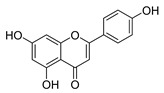	*S. aureus* *B. subtilis* *E. coli* *P. aeruginosa.*	1. Inhibits peptidoglycan synthesis 2. Increases cell membrane permeability	[55]
	morin	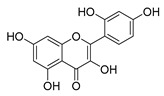	*E. coli*	Inhibition of ATP synthetase	[56]
	silymarin	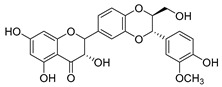	*E. coli*	Inhibition of ATP synthetase	[56]
	epigallocatechin gallate	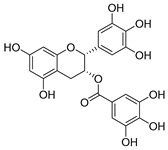	*S. maltophilia*	Inhibits dihydrofolate reductase	[57]
	quercetin	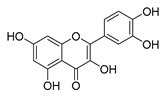	*P. aeruginosa*	1. Inhibits viral polymerase and viral nucleic acid 2. Inhibits the formation of its biofilm	[58]
	galangin	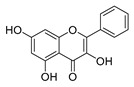	*S. aureus*	1. Destroys the plasma membrane 2. Weakens the cell wall	[59]
	catechin	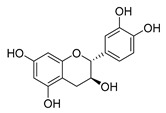	*B. subtilis* *E. coli*	Inhibits the bacterial DNA gyrase	[60] [61]
	baicalin	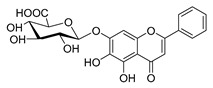	*Salmonella* spp. *Staphylococcus* spp.	Inhibits biofilm formation	[62] [63]
	phloretin	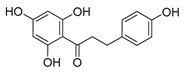	*C. albicans*	1. Inhibits the pathogenicity 2. Inhibits virulence factors	[64]
	silybin	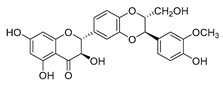	MRSA strain	Inhibits the efflux pump	[65]

## Data Availability

Not applicable.

## References

[1] Reinhardt U., Cheng T. (2000). The world health report 2000–Health systems: Improving performance. Bull. World Health Organ..

[2] Mohr K.I. (2016). History of Antibiotics Research. Curr. Top Microbiol. Immunol..

[3] Ali S.M., Siddiqui R., Khan N.A. (2018). Antimicrobial discovery from natural and unusual sources. J. Pharm. Pharmacol..

[4] Fauci A.S. (2001). Infectious diseases: Considerations for the 21st century. Clin. Infect. Dis..

[5] Keita K., Darkoh C., Okafor F. (2022). Secondary plant metabolites as potent drug candidates against antimicrobial-resistant pathogens. SN Appl. Sci..

[6] Davies S.C., Fowler T., Watson J., Livermore D.M., Walker D. (2013). Annual Report of the Chief Medical Officer: Infection and the rise of antimicrobial resistance. Lancet.

[7] Jia H., Li L., Li W., Hou T., Ma H., Yang Y., Wu A., Liu Y., Wen J., Yang H. (2019). Impact of Healthcare-Associated Infections on Length of Stay: A Study in 68 Hospitals in China. Biomed. Res. Int..

[8] Porras G., Chassagne F., Lyles J.T., Marquez L., Dettweiler M., Salam A.M., Samarakoon T., Shabih S., Farrokhi D.R., Quave C.L. (2021). Ethnobotany and the Role of Plant Natural Products in Antibiotic Drug Discovery. Chem. Rev..

[9] Tiwari P., Khare T., Shriram V., Bae H., Kumar V. (2021). Plant synthetic biology for producing potent phyto-antimicrobials to combat antimicrobial resistance. Biotechnol. Adv..

[10] Vaou N., Stavropoulou E., Voidarou C., Tsigalou C., Bezirtzoglou E. (2021). Towards Advances in Medicinal Plant Antimicrobial Activity: A Review Study on Challenges and Future Perspectives. Microorganisms.

[11] Gonzalez-Lamothe R., Mitchell G., Gattuso M., Diarra M.S., Malouin F., Bouarab K. (2009). Plant antimicrobial agents and their effects on plant and human pathogens. Int. J. Mol. Sci..

[12] Cowan M.M. (1999). Plant products as antimicrobial agents. Clin. Microbiol. Rev..

[13] Savoia D. (2012). Plant-derived antimicrobial compounds: Alternatives to antibiotics. Future Microbiol..

[14] Shankar S.R., Rangarajan R., Sarada D.V.L., Kumar C.S. (2010). Evaluation of antibacterial activity and phytochemical screening of *Wrightia tinctoria* L.. Pharmacogn. J..

[15] Li J.M., Feng S.S., Liu X., Jia X., Qiao F.L., Guo J.L., Deng S.S. (2022). Effects of traditional chinese medicine and its active ingredients on drug-resistant bacteria. Front. Pharmacol..

[16] Kannappan A., Sivaranjani M., Srinivasan R., Rathna J., Pandian S.K., Ravi A.V. (2017). Inhibitory efficacy of geraniol on biofilm formation and development of adaptive resistance in *Staphylococcus epidermidis* RP62A. J. Med. Microbiol..

[17] Upadhyay H.C., Dwivedi G.R., Roy S., Sharma A., Darokar M.P., Srivastava S.K. (2014). Phytol derivatives as drug resistance reversal agents. ChemMedChem.

[18] Nhiri M., Mrid R.B., Omari R.E., Bouargalne Y. (2019). New insights into the therapeutic effects of phenolic acids from sorghum seeds. J. Rep. Pharm. Sci..

[19] Sharma A., Biharee A., Kumar A., Jaitak V. (2020). Antimicrobial terpenoids as a potential substitute in overcoming antimicrobial resistance. Curr. Drug. Targets.

[20] Nazzaro F., Fratianni F., De Martino L., Coppola R., De Feo V. (2013). Effect of essential oils on pathogenic bacteria. Pharmaceuticals.

[21] Burt S.A., Reinders R.D. (2003). Antibacterial activity of selected plant essential oils against *Escherichia coli* O157:H7. Lett. Appl. Microbiol..

[22] Mulyaningsih S., Sporer F., Zimmermann S., Reichling J., Wink M. (2010). Synergistic properties of the terpenoids aromadendrene and 1,8-cineole from the essential oil of *Eucalyptus globulus* against antibiotic-susceptible and antibiotic-resistant pathogens. Phytomedicine.

[23] Di Pasqua R., Betts G., Hoskins N., Edwards M., Ercolini D., Mauriello G. (2007). Membrane toxicity of antimicrobial compounds from Essential oils. J. Agric. Food Chem..

[24] Mulat M., Pandita A., Khan F. (2019). Medicinal plant compounds for combating the multi-drug resistant pathogenic bacteria: A Review. Curr. Pharm. Biotechnol..

[25] Niu C., Afre S., Gilbert E.S. (2006). Subinhibitory concentrations of cinnamaldehyde interfere with quorum sensing. Lett. Appl. Microbiol..

[26] Myszka K., Schmidt M.T., Majcher M., Juzwa W., Olkowicz M., Czaczyk K. (2016). Inhibition of quorum sensing-related biofilm of *Pseudomonas fluorescens* KM121 by *Thymus vulgare* essential oil and its major bioactive compounds. Int. Biodeter. Biodegr..

[27] Mittal R.P., Rana A., Jaitak V. (2019). Essential Oils: An impending substitute of synthetic antimicrobial agents to overcome antimicrobial resistance. Curr. Drug Targets.

[28] Ahmad A., Khan A., Yousuf S., Khan L.A., Manzoor N. (2010). Proton translocating ATPase mediated fungicidal activity of eugenol and thymol. Fitoterapia.

[29] Khan I., Bahuguna A., Shukla S., Aziz F., Chauhan A.K., Ansari M.B., Bajpai V.K., Huh Y.S., Kang S.C. (2020). Antimicrobial potential of the food-grade additive carvacrol against uropathogenic *E. coli* based on membrane depolarization, reactive oxygen species generation, and molecular docking analysis. Microb. Pathog..

[30] Domadia P., Swarup S., Bhunia A., Sivaraman J., Dasgupta D. (2007). Inhibition of bacterial cell division protein FtsZ by cinnamaldehyde. Biochem. Pharmacol..

[31] Naz F., Kumar M., Koley T., Sharma P., Haque M.A., Kapil A., Kumar M., Kaur P., Ethayathulla A.S. (2022). Screening of plant-based natural compounds as an inhibitor of FtsZ from *Salmonella typhi* using the computational, biochemical and in vitro cell-based studies. Int. J. Biol. Macromol..

[32] Zhou F., Ji B., Zhang H., Jiang H., Yang Z., Li J., Li J., Ren Y., Yan W. (2007). Synergistic effect of thymol and carvacrol combined with chelators and organic acids against *Salmonella Typhimurium*. J. Food Prot..

[33] Fontanay S., Grare M., Mayer J., Finance C., Duval R.E. (2008). Ursolic, oleanolic and betulinic acids: Antibacterial spectra and selectivity indexes. J. Ethnopharmacol..

[34] Hikal D.M. (2018). Antibacterial Activity of Piperine and Black Pepper Oil. Biosci. Biotechnol. Res. Asia.

[35] Khan I.A., Mirza Z.M., Kumar A., Verma V., Qazi G.N. (2006). Piperine, a phytochemical potentiator of ciprofloxacin against *Staphylococcus aureus*. Antimicrob. Agents Chemother..

[36] Mittal R.P., Jaitak V. (2019). Plant-derived natural alkaloids as new antimicrobial and adjuvant agents in existing antimicrobial therapy. Curr. Drug Targets.

[37] Su F., Wang J. (2018). Berberine inhibits the MexXY-OprM efflux pump to reverse imipenem resistance in a clinical carbapenem-resistant *Pseudomonas aeruginosa* isolate in a planktonic state. Exp. Ther. Med..

[38] Laudadio E., Cedraro N., Mangiaterra G., Citterio B., Mobbili G., Minnelli C., Bizzaro D., Biavasco F., Galeazzi R. (2019). Natural alkaloid berberine activity against *Pseudomonas aeruginosa* MexXY-mediated aminoglycoside resistance: In Silico and in vitro Studies. J. Nat. Prod..

[39] Mujtaba M.A., Akhter M.H., Alam M.S., Ali M.D., Hussain A. (2022). An updated review on therapeutic potential and recent advances in drug delivery of berberine: Current status and future prospect. Curr. Pharm. Biotechnol..

[40] Wei W.Y., Du H.X., Shao C.Y., Zhou H.F., Lu Y.Y., Yu L., Wan H.T., He Y. (2019). Screening of antiviral components of ma huang tang and investigation on the ephedra alkaloids efficacy on influenza virus type A. Front. Pharmacol..

[41] He N., Wang P., Wang P., Ma C., Kang W. (2018). Antibacterial mechanism of chelerythrine isolated from root of *Toddalia asiatica* (Linn) Lam. BMC Complement. Altern. Med..

[42] Prachayasittikul V., Prachayasittikul S., Ruchirawat S., Prachayasittikul V. (2013). 8-Hydroxyquinolines: A review of their metal chelating properties and medicinal applications. Drug Des. Dev. Ther..

[43] Houdkova M., Rondevaldova J., Doskocil I., Kokoska L. (2017). Evaluation of antibacterial potential and toxicity of plant volatile compounds using new broth microdilution volatilization method and modified MTT assay. Fitoterapia.

[44] McMahon J.B., Currens M.J., Gulakowski R.J., Buckheit R.W., Lackman-Smith C., Hallock Y.F., Boyd M.R. (1995). Michellamine B, a novel plant alkaloid, inhibits human immunodeficiency virus-induced cell killing by at least two distinct mechanisms. Antimicrob. Agents Chemother..

[45] Hamoud R., Reichling J., Wink M. (2015). Synergistic antibacterial activity of the combination of the alkaloid sanguinarine with EDTA and the antibiotic streptomycin against multidrug resistant bacteria. J. Pharm. Pharmacol..

[46] Beuria T.K., Santra M.K., Panda D. (2005). Sanguinarine blocks cytokinesis in bacteria by inhibiting FtsZ assembly and bundling. Biochemistry.

[47] Yin S.J., Rao G.X., Wang J., Luo L.Y., He G.H., Wang C.Y., Ma C.Y., Luo X.X., Hou Z., Xu G.L. (2015). Roemerine improves the survival rate of septicemic BALB/c mice by increasing the cell membrane permeability of *Staphylococcus aureus*. PLoS ONE.

[48] Avci F.G., Atas B., Aksoy C.S., Kurpejovic E., Toplan G.G., Gurer C., Guillerminet M., Orelle C., Jault J.M., Akbulut B.S. (2019). Repurposing bioactive aporphine alkaloids as efflux pump inhibitors. Fitoterapia.

[49] Costa R.S., Lins M.O., Le Hyaric M., Barros T.F., Velozo E.S. (2017). In vitro antibacterial effects of Zanthoxylum tingoassuiba root bark extracts and two of its alkaloids against multiresistant *Staphylococcus aureus*. Rev. Bras. Farmacogn..

[50] Guzman J.D., Wube A., Evangelopoulos D., Gupta A., Hufner A., Basavannacharya C., Rahman M.M., Thomaschitz C., Bauer R., McHugh T.D. (2011). Interaction of N-methyl-2-alkenyl-4-quinolones with ATP-dependent MurE ligase of *Mycobacterium tuberculosis*: Antibacterial activity, molecular docking and inhibition kinetics. J. Antimicrob. Chemother..

[51] Hochfellner C., Evangelopoulos D., Zloh M., Wube A., Guzman J.D., McHugh T.D., Kunert O., Bhakta S., Bucar F. (2015). Antagonistic effects of indoloquinazoline alkaloids on antimycobacterial activity of evocarpine. J. Appl. Microbiol..

[52] Celiz G., Daz M., Audisio M.C. (2011). Antibacterial activity of naringin derivatives against pathogenic strains. J. Appl. Microbiol..

[53] Pyrzynska K. (2022). Hesperidin: A Review on Extraction Methods, Stability and Biological Activities. Nutrients.

[54] Babu K.S., Babu T.H., Srinivas P.V., Sastry B.S., Kishore K.H., Murty U.S., Rao J.M. (2005). Synthesis and in vitro study of novel 7-*O*-acyl derivatives of Oroxylin A as antibacterial agents. Bioorg. Med. Chem. Lett..

[55] Liu R., Zhao B., Wang D.E., Yao T.Y., Pang L., Tu Q., Ahmed S.M., Liu J.J., Wang J.Y. (2012). Nitrogen-containing apigenin analogs: Preparation and biological activity. Molecules.

[56] Chinnam N., Dadi P.K., Sabri S.A., Ahmad M., Kabir M.A., Ahmad Z. (2010). Dietary bioflavonoids inhibit *Escherichia coli* ATP synthase in a differential manner. Int. J. Biol. Macromol..

[57] Navarro-Martinez M.D., Navarro-Peran E., Cabezas-Herrera J., Ruiz-Gomez J., Garcia-Canovas F., Rodriguez-Lopez J.N. (2005). Antifolate activity of epigallocatechin gallate against *Stenotrophomonas maltophilia*. Antimicrob. Agents Chemother..

[58] Pejin B., Ciric A., Markovic J.D., Glamoclija J., Nikolic M., Stanimirovic B., Sokovic M. (2015). Quercetin potently reduces biofilm formation of the strain *Pseudomonas aeruginosa* PAO1 in vitro. Curr. Pharm. Biotechnol..

[59] Cushnie T.P., Lamb A.J. (2005). Detection of galangin-induced cytoplasmic membrane damage in *Staphylococcus aureus* by measuring potassium loss. J. Ethnopharmacol..

[60] Gradisar H., Pristovsek P., Plaper A., Jerala R. (2007). Green tea catechins inhibit bacterial DNA gyrase by interaction with its ATP binding site. J. Med. Chem..

[61] Fathima A., Rao J.R. (2016). Selective toxicity of Catechin-a natural flavonoid towards bacteria. Appl. Microbiol. Biotechnol..

[62] Zhao Q., Chen X.Y., Martin C. (2016). *Scutellaria baicalensis*, the golden herb from the garden of Chinese medicinal plants. Sci. Bull..

[63] Wang J., Zhu J., Meng J., Qiu T., Wang W., Wang R., Liu J. (2021). Baicalin inhibits biofilm formation by influencing primary adhesion and aggregation phases in *Staphylococcus saprophyticus*. Vet. Microbiol..

[64] Liu N., Zhang N., Zhang S., Zhang L., Liu Q. (2021). Phloretin inhibited the pathogenicity and virulence factors against *Candida albicans*. Bioengineered.

[65] Wang D., Xie K., Zou D., Meng M., Xie M. (2018). Inhibitory effects of silybin on the efflux pump of methicillin-resistant *Staphylococcus aureus*. Mol. Med. Rep..

[66] Yin Z., Dickschat J.S. (2022). Engineering fungal terpene biosynthesis. Nat. Prod. Rep..

[67] Bergman M.E., Davis B., Phillips M.A. (2019). Medically useful plant terpenoids: Biosynthesis, occurrence, and mechanism of action. Molecules.

[68] Saha P., Rahman F.I., Hussain F., Rahman S.M.A., Rahman M.M. (2021). Antimicrobial diterpenes: Recent development from natural sources. Front. Pharmacol..

[69] Tu Y. (2011). The discovery of artemisinin (qinghaosu) and gifts from Chinese medicine. Nat. Med..

[70] Tu Y. (2016). Artemisinin-a gift from traditional Chinese medicine to the world (Nobel Lecture). Angew. Chem. Int. Ed. Engl..

[71] O’Neill P.M., Barton V.E., Ward S.A. (2010). The molecular mechanism of action of artemisinin—The debate continues. Molecules.

[72] Meshnick S.R., Taylor T.E., Kamchonwongpaisan S. (1996). Artemisinin and the antimalarial endoperoxides: From herbal remedy to targeted chemotherapy. Microbiol. Rev..

[73] Klonis N., Crespo-Ortiz M.P., Bottova I., Abu-Bakar N., Kenny S., Rosenthal P.J., Tilley L. (2011). Artemisinin activity against *Plasmodium falciparum* requires hemoglobin uptake and digestion. Proc. Natl. Acad. Sci. USA.

[74] Wen W., Yu R. (2011). Artemisinin biosynthesis and its regulatory enzymes: Progress and perspective. Pharmacogn. Rev..

[75] Schramek N., Wang H., Römisch-Margl W., Keil B., Radykewicz T., Winzenhörlein B., Beerhues L., Bacher A., Rohdich F., Gershenzon J. (2010). Artemisinin biosynthesis in growing plants of *Artemisia annua*. A ^13^CO_2_ study. Phytochemistry.

[76] Bouwmeester H.J., Wallaart T.E., Janssen M.H., van Loo B., Jansen B.J., Posthumus M.A., Schmidt C.O., De Kraker J.W., König W.A., Franssen M.C. (1999). Amorpha-4,11-diene synthase catalyses the first probable step in artemisinin biosynthesis. Phytochemistry.

[77] Mercke P., Bengtsson M., Bouwmeester H.J., Posthumus M.A., Brodelius P.E. (2000). Molecular cloning, expression, and characterization of amorpha-4,11-diene synthase, a key enzyme of artemisinin biosynthesis in *Artemisia annua* L.. Arch. Biochem. Biophys..

[78] Picaud S., Mercke P., He X., Sterner O., Brodelius M., Cane D.E., Brodelius P.E. (2006). Amorpha-4,11-diene synthase: Mechanism and stereochemistry of the enzymatic cyclization of farnesyl diphosphate. Arch. Biochem. Biophys..

[79] Kim S.H., Heo K., Chang Y.J., Park S.H., Rhee S.K., Kim S.U. (2006). Cyclization mechanism of amorpha-4,11-diene synthase, a key enzyme in artemisinin biosynthesis. J. Nat. Prod..

[80] Teoh K.H., Polichuk D.R., Reed D.W., Nowak G., Covello P.S. (2006). *Artemisia annua* L. (Asteraceae) trichome-specific cDNAs reveal CYP71AV1, a cytochrome P450 with a key role in the biosynthesis of the antimalarial sesquiterpene lactone artemisinin. FEBS Lett..

[81] Paddon C.J., Westfall P.J., Pitera D.J., Benjamin K., Fisher K., McPhee D., Leavell M.D., Tai A., Main A., Eng D. (2013). High-level semi-synthetic production of the potent antimalarial artemisinin. Nature.

[82] Teoh K., Polichuk D., Reed D., Covello P. (2009). Molecular cloning of an aldehyde dehydrogenase implicated in artemisinin biosynthesis in *Artemisia annua*. Botany.

[83] Zhang Y., Teoh K.H., Reed D.W., Maes L., Goossens A., Olson D.J., Ross A.R., Covello P.S. (2008). The molecular cloning of artemisinic aldehyde Δ11(13) reductase and its role in glandular trichome-dependent biosynthesis of artemisinin in *Artemisia annua*. J. Biol. Chem..

[84] Bertea C.M., Freije J.R., van der Woude H., Verstappen F.W., Perk L., Marquez V., De Kraker J.W., Posthumus M.A., Jansen B.J., de Groot A. (2005). Identification of intermediates and enzymes involved in the early steps of artemisinin biosynthesis in *Artemisia annua*. Planta Med..

[85] Wallaart T.E., Pras N., Quax W.J. (1999). Isolation and identification of dihydroartemisinic acid hydroperoxide from *Artemisia annua*: A novel biosynthetic precursor of artemisinin. J. Nat. Prod..

[86] Sy L.-K., Brown G.D. (2002). The mechanism of the spontaneous autoxidation of dihydroartemisinic acid. Tetrahedron.

[87] Arsenault P.R., Wobbe K.K., Weathers P.J. (2008). Recent advances in artemisinin production through heterologous expression. Curr. Med. Chem..

[88] Zhang Y.S., Liu B.Y., Li Z.Q., Ye H.C., Wang H., Li G.F., Han J.L. (2004). Molecular cloning of a classical plant peroxidase from *Artemisia annua* and its effect on the biosynthesis of artemisinin in vitro. Acta Bot. Sin..

[89] Brown G.D., Sy L.-K. (2007). In vivo transformations of artemisinic acid in *Artemisia annua* plants. Tetrahedron.

[90] Brown G.D., Sy L.-K. (2004). In vivo transformations of dihydroartemisinic acid in *Artemisia annua* plants. Tetrahedron.

[91] Paddon C.J., Keasling J.D. (2014). Semi-synthetic artemisinin: A model for the use of synthetic biology in pharmaceutical development. Nat. Rev. Microbiol..

[92] Martin V.J.J., Pitera D.J., Withers S.T., Newman J.D., Keasling J.D. (2003). Engineering a mevalonate pathway in *Escherichia coli* for production of terpenoids. Nat. Biotechnol..

[93] Martin V.J., Yoshikuni Y., Keasling J.D. (2001). The in vivo synthesis of plant sesquiterpenes by *Escherichia coli*. Biotechnol. Bioeng..

[94] Newman J.D., Marshall J., Chang M., Nowroozi F., Paradise E., Pitera D., Newman K.L., Keasling J.D. (2006). High-level production of amorpha-4,11-diene in a two-phase partitioning bioreactor of metabolically engineered *Escherichia coli*. Biotechnol. Bioeng..

[95] Chang M.C., Keasling J.D. (2006). Production of isoprenoid pharmaceuticals by engineered microbes. Nat. Chem. Biol..

[96] Ro D.K., Paradise E.M., Ouellet M., Fisher K.J., Newman K.L., Ndungu J.M., Ho K.A., Eachus R.A., Ham T.S., Kirby J. (2006). Production of the antimalarial drug precursor artemisinic acid in engineered yeast. Nature.

[97] Jimenez-Arellanes A., Meckes M., Torres J., Luna-Herrera J. (2007). Antimycobacterial triterpenoids from *Lantana hispida* (Verbenaceae). J. Ethnopharmacol..

[98] Kozai K., Suzuki J., Okada M., Nagasaka N. (1999). Effect of oleanolic acid-cyclodextrin inclusion compounds on dental caries by in vitro experiment and rat-caries model. Microbios.

[99] Horiuchi K., Shiota S., Hatano T., Yoshida T., Kuroda T., Tsuchiya T. (2007). Antimicrobial activity of oleanolic acid from *Salvia officinalis* and related compounds on vancomycin-resistant enterococci (VRE). Biol. Pharm. Bull..

[100] Castellano J.M., Ramos-Romero S., Perona J.S. (2022). Oleanolic Acid: Extraction, Characterization and Biological Activity. Nutrients.

[101] Wolska K., Grudniak A.M., Fiecek B., Kraczkiewicz-Dowjat A., Kurek A. (2010). Antibacterial activity of oleanolic and ursolic acids and their derivatives. Open Life Sci..

[102] Pollier J., Goossens A. (2012). Oleanolic acid. Phytochemistry.

[103] Phillips D.R., Rasbery J.M., Bartel B., Matsuda S.P. (2006). Biosynthetic diversity in plant triterpene cyclization. Curr. Opin. Plant Biol..

[104] Abe I. (2007). Enzymatic synthesis of cyclic triterpenes. Nat. Prod. Rep..

[105] Kushiro T., Shibuya M., Ebizuka Y. (1998). *β*-amyrin synthase—Cloning of oxidosqualene cyclase that catalyzes the formation of the most popular triterpene among higher plants. Eur. J. Biochem..

[106] Abe I., Rohmer M., Prestwich G.D. (1993). Enzymatic Cyclization of Squalene and Oxidosqualene to Sterols and Triterpenes. Chem. Rev..

[107] Seo S., Yoshimura Y., Uomori A., Takeda K., Seto H., Ebizuka Y., Sankawa U. (1988). Biosynthesis of triterpenes, ursolic acid and oleanolic acid in tissue cultures of *Rabdosia japonica* Hara fed [5-^13^C^2^H_2_] mevalonolactone and [2-^13^C^2^H_3_] acetate. J. Am. Chem. Soc..

[108] Fukushima E.O., Seki H., Ohyama K., Ono E., Umemoto N., Mizutani M., Saito K., Muranaka T. (2011). CYP716A subfamily members are multifunctional oxidases in triterpenoid biosynthesis. Plant Cell Physiol..

[109] Carelli M., Biazzi E., Panara F., Tava A., Scaramelli L., Porceddu A., Graham N., Odoardi M., Piano E., Arcioni S. (2011). *Medicago truncatula* CYP716A12 is a multifunctional oxidase involved in the biosynthesis of hemolytic saponins. Plant Cell.

[110] Han Y., Sun Z., Chen W. (2019). Antimicrobial Susceptibility and Antibacterial Mechanism of Limonene against Listeria monocytogenes. Molecules.

[111] Cheng S., Liu X., Jiang G., Wu J., Zhang J.L., Lei D., Yuan Y.J., Qiao J., Zhao G.R. (2019). Orthogonal Engineering of Biosynthetic Pathway for Efficient Production of Limonene in *Saccharomyces cerevisiae*. ACS Synth. Biol..

[112] Parthasarathy A., Borrego E.J., Savka M.A., Dobson R.C.J., Hudson A.O. (2021). Amino acid-derived defense metabolites from plants: A potential source to facilitate novel antimicrobial development. J. Biol. Chem..

[113] Othman L., Sleiman A., Abdel-Massih R.M. (2019). Antimicrobial activity of polyphenols and alkaloids in middle eastern plants. Front. Microbiol..

[114] Cushnie T.P.T., Cushnie B., Lamb A.J. (2014). Alkaloids: An overview of their antibacterial, antibiotic-enhancing and antivirulence activities. Int. J. Antimicrob. Agents.

[115] Thawabteh A., Juma S., Bader M., Karaman D., Scrano L., Bufo S.A., Karaman R. (2019). The biological activity of natural alkaloids against herbivores, cancerous cells and pathogens. Toxins.

[116] Khameneh B., Iranshahy M., Ghandadi M., Atashbeyk D.G., Bazzaz B.S.F., Iranshahi M. (2015). Investigation of the antibacterial activity and efflux pump inhibitory effect of co-loaded piperine and gentamicin nanoliposomes in methicillin-resistant *Staphylococcus aureus*. Drug Dev. Ind. Pharm..

[117] Mabhiza D., Chitemerere T., Mukanganyama S. (2016). Antibacterial Properties of Alkaloid Extracts from *Callistemon citrinus* and *Vernonia adoensis* against *Staphylococcus aureus* and *Pseudomonas aeruginosa*. Int. J. Med. Chem..

[118] Wink M., Ashour M.L., El-Readi M.Z. (2012). Secondary metabolites from plants inhibiting ABC transporters and reversing resistance of cancer cells and microbes to cytotoxic and antimicrobial agents. Front. Microbiol..

[119] Gibbons S., Udo E.E. (2000). The effect of reserpine, a modulator of multidrug efflux pumps, on the *in vitro* activity of tetracycline against clinical isolates of methicillin resistant *Staphylococcus aureus* (MRSA) possessing the tet (K) determinant. Phytother. Res..

[120] Stermitz F.R., Lorenz P., Tawara J.N., Zenewicz L.A., Lewis K. (2000). Synergy in a medicinal plant: Antimicrobial action of berberine potentiated by 5’-methoxyhydnocarpin, a multidrug pump inhibitor. Proc. Natl. Acad. Sci. USA.

[121] Ahmed M., Borsch C.M., Neyfakh A.A., Schuldiner S. (1993). Mutants of the Bacillus subtilis multidrug transporter Bmr with altered sensitivity to the antihypertensive alkaloid reserpine. J. Biol. Chem..

[122] Xie Q., Johnson B.R., Wenckus C.S., Fayad M.I., Wu C.D. (2012). Efficacy of berberine, an antimicrobial plant alkaloid, as an endodontic lrrigant against a mixed-culture biofilm in an in vitro tooth model. J. Endodont..

[123] Yi Z.B., Yan Y., Liang Y.Z., Bao Z. (2007). Evaluation of the antimicrobial mode of berberine by LC/ESI-MS combined with principal component analysis. J. Pharm. Biomed. Anal..

[124] Albert A., Magrath D. (1947). The choice of a chelating agent for inactivating trace metals: II. Derivatives of oxine (8-hydroxyquinoline). Biochem. J..

[125] Morita Y., Nakashima K., Nishino K., Kotani K., Tomida J., Inoue M., Kawamura Y. (2016). Berberine is a novel type efflux inhibitor which attenuates the MexXY-mediated aminoglycoside resistance in *Pseudomonas aeruginosa*. Front. Microbiol..

[126] Tegos G., Stermitz F.R., Lomovskaya O., Lewis K. (2002). Multidrug pump inhibitors uncover remarkable activity of plant antimicrobials. Antimicrob. Agents Chemother..

[127] Das S., Kumar G.S., Ray A., Maiti M. (2003). Spectroscopic and thermodynamic studies on the binding of sanguinarine and berberine to triple and double helical DNA and RNA structures. J. Biomol. Struct. Dyn..

[128] Bhadra K., Maiti M., Kumar G.S. (2008). Berberine-DNA complexation: New insights into the cooperative binding and energetic aspects. Biochim. Biophys. Acta..

[129] Eckhardt K., Zeller K.P., Siehl H.U., Berger S., Sicker D. (2017). Look, the Yellow is so near: Berberine chloride from berberine bark. Chem. Unserer. Zeit.

[130] He S.M., Liang Y.L., Cong K., Chen G., Zhao X., Zhao Q.M., Zhang J.J., Wang X., Dong Y., Yang J.L. (2018). Identification and characterization of genes involved in benzylisoquinoline alkaloid biosynthesis in *Coptis* species. Front. Plant Sci..

[131] Samanani N., Facchini P.J. (2002). Purification and characterization of norcoclaurine synthase. The first committed enzyme in benzylisoquinoline alkaloid biosynthesis in plants. J. Biol. Chem..

[132] Samanani N., Liscombe D.K., Facchini P.J. (2004). Molecular cloning and characterization of norcoclaurine synthase, an enzyme catalyzing the first committed step in benzylisoquinoline alkaloid biosynthesis. Plant J..

[133] Minami H., Dubouzet E., Iwasa K., Sato F. (2007). Functional analysis of norcoclaurine synthase in *Coptis japonica*. J. Biol. Chem..

[134] Rueffer M., Nagakura N., Zenk M.H. (1983). Partial purification and properties of *S*-adenosylmethionine: (*R*), (*S*)-Norlaudanosoline-6-*O*-methyltransferase from *Argemone platyceras* cell cultures. Planta Med..

[135] Sato F., Tsujita T., Katagiri Y., Yoshida S., Yamada Y. (1994). Purification and characterization of *S*-adenosyl-L-methionine: Norcoclaurine 6-*O*-methyltransferase from cultured *Coptis japonica* cells. Eur. J. Biochem..

[136] Loeffler S., Deusneumann B., Zenk M.H. (1995). *S*-adenosyl-L-methionine-(*S*)-coclaurine-*N*-methyltransferase from *Tinospora-Cordifolia*. Phytochemistry.

[137] Choi K.B., Morishige T., Shitan N., Yazaki K., Sato F. (2002). Molecular cloning and characterization of coclaurine *N*-methyltransferase from cultured cells of *Coptis japonica*. J. Biol. Chem..

[138] Morishige T., Tsujita T., Yamada Y., Sato F. (2000). Molecular characterization of the *S*-adenosyl-L-methionine: 3′-hydroxy-*N*-methylcoclaurine 4′-*O*-methyltransferase involved in isoquinoline alkaloid biosynthesis in *Coptis japonica*. J. Biol. Chem..

[139] Frenzel T., Zenk M.H. (1990). *S*-Adenosyl-L-Methionine—3′-Hydroxy-*N*-Methyl-(*S*)-Coclaurine-4′-*O*-Methyl Transferase, a Regioselective and Stereoselective Enzyme of the (*S*)-Reticuline Pathway. Phytochemistry.

[140] Chapple C. (1998). Molecular-genetic analysis of plant cytochrome P450-dependent monooxygenases. Annu. Rev. Plant Physiol. Plant Mol. Biol..

[141] Mizutani M., Sato F. (2011). Unusual P450 reactions in plant secondary metabolism. Arch. Biochem. Biophys..

[142] Ikezawa N., Tanaka M., Nagayoshi M., Shinkyo R., Sakaki T., Inouye K., Sato F. (2003). Molecular cloning and characterization of CYP719, a methylenedioxy bridge-forming enzyme that belongs to a novel P450 family, from cultured *Coptis japonica* cells. J. Biol. Chem..

[143] Kutchan T.M., Dittrich H. (1995). Characterization and mechanism of the berberine bridge enzyme, a covalently flavinylated oxidase of benzophenanthridine alkaloid biosynthesis in plants. J. Biol. Chem..

[144] Xu Z.X., Xia L.Q., Sun M.S., Huang P., Zeng J.G. (2022). Effects of codon optimization, *N*-terminal truncation and gene dose on the heterologous expression of berberine bridge enzyme. World J. Microb. Biot..

[145] Muemmler S., Rueffer M., Nagakura N., Zenk M.H. (1985). *S*-adenosyl-L-methionine: (*S*)-scoulerine 9-*O*-methyltransferase, a highly stereo- and regio-specific enzyme in tetrahydroprotoberberine biosynthesis. Plant Cell Rep..

[146] Takeshita N., Fujiwara H., Mimura H., Fitchen J.H., Yamada Y., Sato F. (1995). Molecular cloning and characterization of *S*-adenosyl-L-methionine: Scoulerine-9-*O*-methyltransferase from cultured cells of *Coptis japonica*. Plant Cell Physiol..

[147] Galneder E., Rueffer M., Wanner G., Tabata M., Zenk M.H. (1988). Alternative final steps in berberine biosynthesis in *Coptis japonica* cell cultures. Plant Cell Rep..

[148] Okada N., Koizumi N., Tanaka T., Ohkubo H., Nakanishi S., Yamada Y. (1989). Isolation, sequence, and bacterial expression of a cDNA for (*S*)-tetrahydroberberine oxidase from cultured berberine-producing *Coptis japonica* cells. Proc. Natl. Acad. Sci. USA.

[149] Payne J.T., Valentic T.R., Smolke C.D. (2021). Complete biosynthesis of the bisbenzylisoquinoline alkaloids guattegaumerine and berbamunine in yeast. Proc. Natl. Acad. Sci. USA.

[150] Hawkins K.M., Smolke C.D. (2008). Production of benzylisoquinoline alkaloids in *Saccharomyces cerevisiae*. Nat. Chem. Biol..

[151] Park S.U., Facchini P.J. (2000). *Agrobacterium rhizogenes*-mediated transformation of opium poppy, *Papaver somniferum* L., and California poppy, *Eschscholzia californica* cham., root cultures. J. Exp. Bot..

[152] Sivakumar G. (2018). Upstream biomanufacturing of pharmaceutical colchicine. Crit. Rev. Biotechnol..

[153] Jana S., Shekhawat G.S. (2010). Critical review on medicinally potent plant species: *Gloriosa superba*. Fitoterapia.

[154] Slobodnick A., Shah B., Pillinger M.H., Krasnokutsky S. (2015). Colchicine: Old and New. Am. J. Med..

[155] Karamanou M., Tsoucalas G., Pantos K., Androutsos G. (2018). Isolating Colchicine in 19th Century: An old drug revisited. Curr. Pharm. Des..

[156] Zhang F.S., He Q.Z., Qin C.H., Little P.J., Weng J.P., Xu S.W. (2022). Therapeutic potential of colchicine in cardiovascular medicine: A pharmacological review. Acta. Pharmacol. Sin..

[157] Bhattacharyya B., Panda D., Gupta S., Banerjee M. (2008). ChemInform Abstract: Anti-mitotic activity of colchicine and the structural basis for its interaction with tubulin. ChemInform.

[158] Chiu L., Lo C.H., Shen M., Chiu N., Aggarwal R., Lee J., Choi Y.G., Lam H., Prsic E.H., Chow R. (2021). Colchicine use in patients with COVID-19: A systematic review and meta-analysis. PLoS ONE.

[159] Leete E., Nemeth P.E. (1960). The Biogenesis of the Alkaloids of *Colchicum*. I. The incorporation of phenylalanine into colchicine. J. Am. Chem. Soc..

[160] Herbert R.B. (2003). The biosynthesis of plant alkaloids and nitrogenous microbial metabolites. Nat. Prod. Rep..

[161] Battersby A.R., Herbert R.B., McDonald E., Ramage R., Clements J.H. (1972). Alkaloid biosynthesis. 18. Biosynthesis of colchicine from the 1-phenethylisoquinoline system. J. Chem. Soc. Perkin. 1.

[162] Herbert R.B., Kattah A.E., Knagg E. (1990). The Biosynthesis of the phenethylisoquinoline alkaloid colchicine. Early and intermediate stages. Tetrahedron.

[163] Nasreen A., Gundlach H., Zenk M.H. (1997). Incorporation of phenethylisoquinolines into colchicine in isolated seeds of *Colchicum autumnale*. Phytochemistry.

[164] Leete E. (1965). Biosynthesis of the tropolone ring of colchicine. Tetrahedron Lett..

[165] Herbert R.B., Knagg E. (1986). The biosynthesis of the phenethylisoquinoline alkaloid, colchicine, from cinnamaldehyde and dihydrocinnamaldehyde. Tetrahedron Lett..

[166] Maier U.H., Zenk M.H. (1997). Colchicine is formed by *para-para* phenol coupling from autumnaline. Tetrahedron Lett..

[167] Rueffer M., Zenk M.H. (1998). Microsome-mediated transformation of *O*-methylandrocymbine to demecolcine and colchicine. FEBS Lett..

[168] Sheldrake P.W., Suckling K.E., Woodhouse R.N., Murtagh A.J. (1998). Biosynthesis Part 30.1 Colchicine: Studies on the ring expansion step focusing on the fate of the hydrogens at C-4 of autumnaline. J. Chem. Soc. Perkin Trans. 1.

[169] Nett R.S., Lau W., Sattely E.S. (2020). Discovery and engineering of colchicine alkaloid biosynthesis. Nature.

[170] Nett R.S., Sattely E.S. (2021). Total biosynthesis of the tubulin-binding alkaloid colchicine. J. Am. Chem. Soc..

[171] Maeda H., Dudareva N. (2012). The shikimate pathway and aromatic amino acid biosynthesis in plants. Annu. Rev. Plant Biol..

[172] Sunnadeniya R., Bean A., Brown M., Akhavan N., Hatlestad G., Gonzalez A., Symonds V.V., Lloyd A. (2016). Tyrosine Hydroxylation in betalain pigment biosynthesis is performed by cytochrome P450 enzymes in beets (*Beta vulgaris*). PLoS ONE.

[173] Luk L.Y.P., Bunn S., Liscombe D.K., Facchini P.J., Tanner M.E. (2007). Mechanistic studies on norcoclaurine synthase of benzylisoquinoline alkaloid biosynthesis: An enzymatic Pictet-Spengler reaction. Biochemistry.

[174] Ruff B.M., Brase S., O’Connor S.E. (2012). Biocatalytic production of tetrahydroisoquinolines. Tetrahedron Lett..

[175] Nishihachijo M., Hirai Y., Kawano S., Nishiyama A., Minami H., Katayama T., Yasohara Y., Sato F., Kumagai H. (2014). Asymmetric synthesis of tetrahydroisoquinolines by enzymatic Pictet-Spengler reaction. Biosci. Biotech. Bioch..

[176] Barker A.C., Julian D.R., Ramage R., Woodhouse R.N., Hardy G., McDonald E., Battersby A.R. (1998). Biosynthesis. Part 28.^1,2^ Colchicine: Definition of intermediates between *O*-mehylandrocymbine and colchicine and studies on speciosine. J. Chem. Soc. Perk Trans..

[177] McDonald E., Ramage R., Woodhouse R.N., Underhill E.W., Wetter L.R., Battersby A.R. (1998). Biosynthesis. Part 27.^1,2^ Colchicine: Studies of the phenolic oxidative coupling and ring-expansion processes based on incorporation of multiply labelled 1-phenethylisoquinolines. J. Chem. Soc. Perk Trans..

[178] Shang X.F., Morris-Natschke S.L., Liu Y.Q., Guo X., Xu X.S., Goto M., Li J.C., Yang G.Z., Lee K.H. (2018). Biologically active quinoline and quinazoline alkaloids part I. Med. Res. Rev..

[179] Subramani R., Narayanasamy M., Feussner K.D. (2017). Plant-derived antimicrobials to fight against multi-drug-resistant human pathogens. 3 Biotech.

[180] Casciaro B., Mangiardi L., Cappiello F., Romeo I., Loffredo M.R., Iazzetti A., Calcaterra A., Goggiamani A., Ghirga F., Mangoni M.L. (2020). Naturally-Occurring alkaloids of plant origin as potential antimicrobials against antibiotic-resistant infections. Molecules.

[181] Mitchell G., Gattuso M., Grondin G., Marsault E., Bouarab K., Malouin F. (2011). Tomatidine Inhibits Replication of *Staphylococcus aureus* Small-Colony Variants in Cystic Fibrosis Airway Epithelial Cells. Antimicrob. Agents Chemother..

[182] Dorsaz S., Snaka T., Favre-Godal Q., Maudens P., Boulens N., Furrer P., Ebrahimi S.N., Hamburger M., Allemann E., Gindro K. (2017). Identification and mode of action of a plant natural product targeting human fungal pathogens. Antimicrob. Agents Chemother..

[183] Akiyama R., Lee J.H., Nakayasu M., Osakabe K., Osakabe Y., Umemoto N., Saito K., Muranaka T., Sugimoto Y., Mizutani M. (2019). Characterization of steroid 5α-reductase involved in *α*-tomatine biosynthesis in tomatoes. Plant Biotechnol..

[184] Abdel-Motaal F.F., Nassar M.S.M., El-Zayat S.A., El-Sayed M.A., Ito S. (2009). Responses of fungi to tropane alkaloids produced by a medicinal plant *Hyoscyamus muticus* (Egyptian Henbane). Folia Microbiol..

[185] Abdel-Motaal F.F., El-Zayat S.A., Kosaka Y., El-Sayed M.A., Kashima R., Maeda Y., Nassar M.S.M., Ito S. (2010). Antifungal activities of hyoscyamine and scopolamine against two major rice pathogens: *Magnaporthe oryzae* and *Rhizoctonia solani*. J. Gen. Plant Pathol..

[186] Srinivasan P., Smolke C.D. (2020). Biosynthesis of medicinal tropane alkaloids in yeast. Nature.

[187] Davison E.K., Brimble M.A. (2019). Natural product derived privileged scaffolds in drug discovery. Curr. Opin. Chem. Biol..

[188] Shen N., Wang T.F., Gan Q., Liu S., Wang L., Jin B. (2022). Plant flavonoids: Classification, distribution, biosynthesis, and antioxidant activity. Food Chem..

[189] Chiu H.H., Hsieh Y.C., Chen Y.H., Wang H.Y., Lu C.Y., Chen C.J., Li Y.K. (2016). Three important amino acids control the regioselectivity of flavonoid glucosidation in glycosyltransferase-1 from *Bacillus cereus*. Appl. Microbiol. Biotechnol..

[190] Roy A., Khan A., Ahmad I., Alghamdi S., Rajab B.S., Babalghith A.O., Alshahrani M.Y., Islam S., Islam M.R. (2022). Flavonoids a bioactive compound from medicinal plants and its therapeutic applications. Biomed. Res. Int..

[191] Li Y., Kumar P.S., Tan S., Huang C., Xiang Z., Qiu J., Tan X., Luo J., He M. (2022). Anticancer and antibacterial flavonoids from the callus of *Ampelopsis grossedentata*; a new weapon to mitigate the proliferation of cancer cells and bacteria. RSC Adv..

[192] Di Pierro F., Iqtadar S., Khan A., Mumtaz S.U., Chaudhry M.M., Bertuccioli A., Derosa G., Maffioli P., Togni S., Riva A. (2021). Potential clinical benefits of quercetin in the early stage of COVID-19: Results of a second, pilot, randomized, controlled and open-label clinical trial. Int. J. Gen. Med..

[193] Biharee A., Sharma A., Kumar A., Jaitak V. (2020). Antimicrobial flavonoids as a potential substitute for overcoming antimicrobial resistance. Fitoterapia.

[194] Xie Y.X., Yang W.J., Tang F., Chen X.Q., Ren L.C. (2015). Antibacterial activities of flavonoids: Structure-activity relationship and mechanism. Curr. Med. Chem..

[195] Smejkal K., Chudík S., Kloucek P., Marek R., Cvacka J., Urbanová M., Julínek O., Kokoska L., Slapetová T., Holubová P. (2008). Antibacterial *C*-geranylflavonoids from *Paulownia tomentosa* Fruits. J. Nat. Prod..

[196] Tran T.D., Do T.H., Tran N.C., Ngo T.D., Huynh T.N., Tran C.D., Thai K.M. (2012). Synthesis and anti Methicillin resistant *Staphylococcus aureus* activity of substituted chalcones alone and in combination with non-beta-lactam antibiotics. Bioorg. Med. Chem. Lett..

[197] Rütschlin S., Böttcher T. (2020). Inhibitors of Bacterial Swarming Behavior. Chemistry.

[198] Austin M.B., Noel J.P. (2003). The chalcone synthase superfamily of type III polyketide synthases. Nat. Prod. Rep..

[199] Reimold U., Kröger M., Kreuzaler F., Hahlbrock K. (1983). Coding and 3′ non-coding nucleotide sequence of chalcone synthase mRNA and assignment of amino acid sequence of the enzyme. Embo. J..

[200] Pandith S.A., Ramazan S., Khan M.I., Reshi Z.A., Shah M.A. (2019). Chalcone synthases (CHSs): The symbolic type III polyketide synthases. Planta.

[201] Ferrer J.L., Jez J.M., Bowman M.E., Dixon R.A., Noel J.P. (1999). Structure of chalcone synthase and the molecular basis of plant polyketide biosynthesis. Nat. Struct. Biol..

[202] Jones J.A., Vernacchio V.R., Sinkoe A.L., Collins S.M., Ibrahim M.H.A., Lachance D.M., Hahn J., Koffas M.A.G. (2016). Experimental and computational optimization of an *Escherichia coli* co-culture for the efficient production of flavonoids. Metab. Eng..

[203] Zhou S., Lyu Y., Li H., Koffas M.A.G., Zhou J. (2019). Fine-tuning the (2S)-naringenin synthetic pathway using an iterative high-throughput balancing strategy. Biotechnol. Bioeng..

[204] Zhao Q., Zhang Y., Wang G., Hill L., Weng J.K., Chen X.Y., Xue H., Martin C. (2016). A specialized flavone biosynthetic pathway has evolved in the medicinal plant, *Scutellaria baicalensis*. Sci. Adv..

[205] Kitamura K., Honda M., Yoshizaki H., Yamamoto S., Nakane H., Fukushima M., Ono K., Tokunaga T. (1998). Baicalin, an inhibitor of HIV-1 production in vitro. Antiviral Res..

[206] Li B.Q., Fu T., Dongyan Y., Mikovits J.A., Ruscetti F.W., Wang J.M. (2000). Flavonoid baicalin inhibits HIV-1 infection at the level of viral entry. Biochem. Biophys. Res. Commun..

[207] Liu W.X., Feng Y., Yu S.H., Fan Z.Q., Li X.L., Li J.Y., Yin H.F. (2021). The flavonoid biosynthesis network in plants. Int. J. Mol. Sci..

[208] Pei T.L., Yan M.X., Li T., Li X.Q., Yin Y.J., Cui M.Y., Fang Y.M., Liu J., Kong Y., Xu P. (2022). Characterization of UDP-glycosyltransferase family members reveals how major flavonoid glycoside accumulates in the roots of *Scutellaria baicalensis*. BMC Genom..

[209] Han H.D., Lee Y.S., Ahn J.H. (2016). Biological synthesis of baicalein derivatives using *Escherichia coli*. J. Microbiol. Biotechnol..

[210] Liao J.J., Xie L., Liu T.Y., Mo C.M., Cui S.R., Jia X.L., Huang X.Y., Luo Z.L., Ma X.J. (2022). Heterologous biosynthesis of health-promoting baicalein in *Lycopersicon esculentum*. Molecules.

[211] Caputi L., Franke J., Farrow S.C., Chung K., Payne R.M.E., Nguyen T.D., Dang T.T., Soares Teto Carqueijeiro I., Koudounas K., Dugé de Bernonville T. (2018). Missing enzymes in the biosynthesis of the anticancer drug vinblastine in *Madagascar periwinkle*. Science.

[212] Zhang J., Hansen L.G., Gudich O., Viehrig K., Lassen L.M.M., Schrübbers L., Adhikari K.B., Rubaszka P., Carrasquer-Alvarez E., Chen L. (2022). A microbial supply chain for production of the anti-cancer drug vinblastine. Nature.

[213] Lau W., Sattely E.S. (2015). Six enzymes from mayapple that complete the biosynthetic pathway to the etoposide aglycone. Science.

